# Comparison of carbohydrate ABC importers from *Mycobacterium tuberculosis*

**DOI:** 10.1186/s12864-021-07972-w

**Published:** 2021-11-19

**Authors:** Lilia I. De la Torre, José G. Vergara Meza, Sindy Cabarca, André G. Costa-Martins, Andrea Balan

**Affiliations:** 1 Department of Microbiology, Institute of Biomedical Science, University of São Paulo, São Paulo, Brazil; 2 Genectics and Molecular Biology Postgraduate Program, Institute of Biology, State University of Campinas, São Paulo, Brazil; 3 Biomedical Research Group, University of Sucre, Sucre, Colombia; 4 Department of Parasitology, Institute of Biomedical Science, University of São Paulo, São Paulo, Brazil; 5 Department of Clinical and Toxicological Analyses, School of Pharmaceutical Sciences, University of São Paulo, São Paulo, Brazil; 6 Laboratory of Applied Structural Biology, Department of Microbiology, Institute of Biomedical Sciences, University of São Paulo, Av. Prof. Lineu Prestes, 1374; Cidade Universitária, São Paulo, Brazil

**Keywords:** Carbohydrate uptake, ABC transporters, Phylogeny, Multitask NBDs, Structure-function, Protein-protein interaction

## Abstract

**Background:**

*Mycobacterium tuberculosis,* the etiological agent of tuberculosis, has at least four ATP-Binding Cassette (ABC) transporters dedicated to carbohydrate uptake: LpqY/SugABC, UspABC, Rv2038c-41c, and UgpAEBC. LpqY/SugABC transporter is essential for *M. tuberculosis* survival in vivo and potentially involved in the recycling of cell wall components. The three-dimensional structures of substrate-binding proteins (SBPs) LpqY, UspC, and UgpB were described, however, questions about how these proteins interact with the cognate transporter are still being explored. Components of these transporters, such as SBPs, show high immunogenicity and could be used for the development of diagnostic and therapeutic tools. In this work, we used a phylogenetic and structural bioinformatics approach to compare the four systems, in an attempt to predict functionally important regions.

**Results:**

Through the analysis of the putative orthologs of the carbohydrate ABC importers in species of *Mycobacterium* genus it was shown that Rv2038c-41c and UgpAEBC systems are restricted to pathogenic species. We showed that the components of the four ABC importers are phylogenetically separated into four groups defined by structural differences in regions that modulate the functional activity or the interaction with domain partners. The regulatory region in nucleotide-binding domains, the periplasmic interface in transmembrane domains and the ligand-binding pocket of the substrate-binding proteins define their substrates and segregation in different branches. The interface between transmembrane domains and nucleotide-binding domains show conservation of residues and charge.

**Conclusions:**

The presence of four ABC transporters in *M. tuberculosis* dedicated to uptake and transport of different carbohydrate sources, and the exclusivity of at least two of them being present only in pathogenic species of *Mycobacterium* genus, highlights their relevance in virulence and pathogenesis. The significant differences in the SBPs, not present in eukaryotes, and in the regulatory region of NBDs can be explored for the development of inhibitory drugs targeting the bacillus. The possible promiscuity of NBDs also contributes to a less specific and more comprehensive control approach.

**Supplementary Information:**

The online version contains supplementary material available at 10.1186/s12864-021-07972-w.

## Background

*Mycobacterium tuberculosis* is the causative agent of tuberculosis, one of the top causes of human death worldwide from a single infectious agent. About a quarter of the world’s population has been infected by *M. tuberculosis* and thus at risk of developing tuberculosis disease [1]. Although there are many studies about the ability of *M. tuberculosis* to persist inside the host cells under a variety of adverse conditions including oxidative stress, hypoxia, and nutrient starvation [2], some aspects of the mechanisms behind are poorly understood. The upregulation of different nutrient uptake responsive genes at different stages of infection indicates that *M. tuberculosis* utilizes a set of nutrient sources from early to persistent phase, that include ions, amino acids, lipids, carbohydrates, and others required for many biological processes [3–5]. Different mechanisms to obtain essential nutrients from microenvironments and the broad range of substrate specificities, allow bacteria to quickly adapt to and colonize challenging environments. Some of these nutrient acquisition mechanisms are described as key virulence determinants used by pathogens to mediate disease [6, 7].

Carbohydrates have traditionally been considered an important source of carbon and energy supply in bacteria. Particularly, *M. tuberculosis* prefers host lipids, as evidenced in an over-representation of genes in the genome that encode enzymes for fatty acid metabolism, and upregulation of such genes during macrophage infection [8, 9]. Even though these studies suggest that lipids are the main source of carbon and energy for *M. tuberculosis,* other yet to be identified carbon sources also have an important role to play [9, 10]. In this sense, *M. tuberculosis* is equipped with five putative importers of carbohydrates: one belonging to the major facilitator superfamily and four members of the ATP-Binding Cassette (ABC) transporter family, one of the largest families of paralogous proteins present in the bacillus [11]. Genes encoding ABC transporters account for about 2.5% of *M. tuberculosis* genome, being reported 20 importers and 14 exporters [12]. In comparison with *Escherichia coli*, *Bacillus subtilis,* and even *Mycobacterium smegmatis*, there is a significant reduction of genes encoding ABC transporter components in *M. tuberculosis* genome, particularly evident for the transporters involved in carbohydrate uptake [13]. ABC transporters dedicated to carbohydrate transport have been related to virulence and pathogenesis in bacteria, but the role of these transporters in *M. tuberculosis* still needs to be explored.

ABC transporters type importers are responsible for the translocation of the substrate into the cell and they were identified until today in prokaryotes. Structurally, they consist of oligo protein assemblies with two hydrophobic transmembrane domains (TMDs) that form the transport channel, two cytoplasmic nucleotide-binding domains (NBDs), which are responsible for the breakdown of ATP and provision of energy for the transport process, and an additional periplasmic substrate-binding protein (SBP) or domain (SBD) exposed to the periplasm of the cell [14].

The four operons that encode ABC transporters dedicated to carbohydrate uptake in *M. tuberculosis* genome are: *lpqY-sugABC, rv2038c-41c, uspABC*, and *ugpAEBC* [12], where *lpqY, rv2041, uspC* and *ugpB* encode the SBPs, SugAB, Rv2039c-40c, UspAB and UgpAE encode the heterodimeric TMDs, and *sugC*, *rv2038c* and *ugpC* encode the NBDs. Genetic and cellular approaches applied to the study of LpqY/SugABC transporter demonstrated that it was essential for virulence of *M. tuberculosis* in vivo and potentially involved in recycling of trehalose monomycolate, a cell wall glycolipid [15]. This transporter also raises interest for the detection of *M. tuberculosis* in sputum samples, since it can probably be the pathway for uptake of a solvatochromic trehalose probe [16]. The three-dimensional structure of the *M. smegmatis* full transporter LpqY/SugABC was resolved in different states evidencing important secondary structures and residues for the trehalose transport mechanism [17]. Additionally, the interaction of the SBP LpqY of *M. tuberculosis* and *M. thermoresistible* with different ligands was also explored [18]. The transporter UspABC consists of the SBP UspC and two TMDs (UspA and UspB) but lacks the NBD domain. The three-dimensional structure of the UspC was solved by X-ray crystallography (PDB codes 5K2X and 5K2Y), and binding studies with different putative substrates showed a higher affinity for carbohydrates containing an amino group at the C2 or C3 position, like D-glucosamine-6-phosphate and chitobiose than s*n*-glycero-3-phosphocholine, D-glucose or α,α-D-trehalose [19]. The UgpAEBC ABC transporter is predicted to be involved in scavenging of glycerophospholipids [20], which are carbon or phosphate sources that could be available for *M. tuberculosis* inside of macrophage or another cell host. The crystal structure of the substrate-binding protein UgpB was resolved in presence of glycerophoscholine (PDB code 6R1B), but functional studies revealed that the protein also could bind other glycerophosphodiesters [21]. The less known *M. tuberculosis* ABC importer is Rv2038c-41c. Studies with the SBP Rv2041c showed increased expression under conditions that are similar to those in a phagocytic environment (low pH and hypoxia) [22]. Immunological studies with a cocktail of five commonly used serological antigens for tuberculosis diagnostic (CFP-10, ESAT-6, HSP-X, Ag85 complex, and PstS1), showed increased sensitivity for TB diagnostic when Rv2041c was added in the mixture, indicating the capability of this protein to induce the cellular immune response, and highlighting its potential for the development of a vaccine candidate against *M. tuberculosis* [23]. Table 1 shows a resume of the most available information for each component.

**Table 1:**
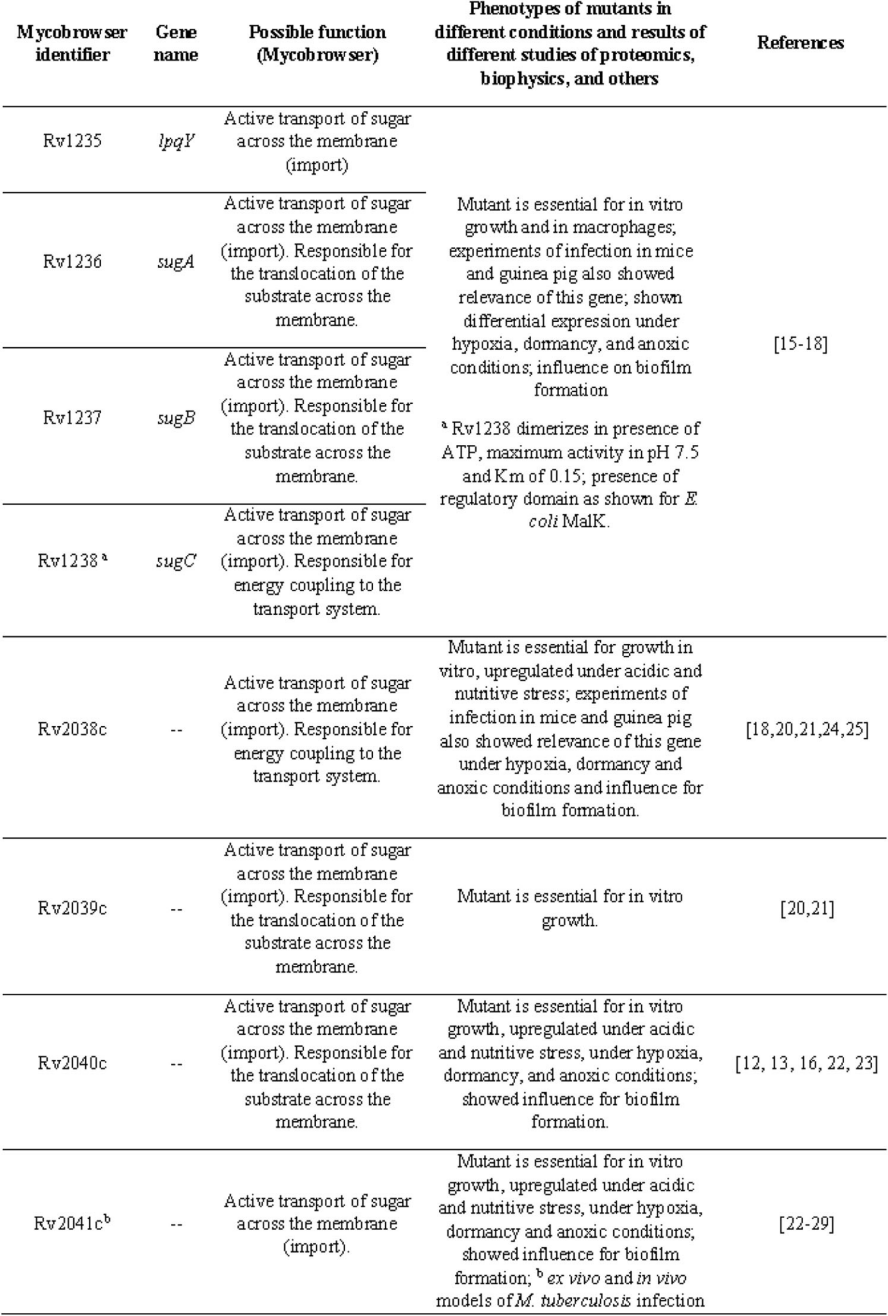
Resume of available data regarding the components of carbohydrate ABC transporters from *Mycobacterium* species

In this work, we made a comprehensive comparative analysis of the four ABC transporters type importer from *M. tuberculosis* involved in carbohydrate uptake. The phylogenetic relationship among the components and their conservation in different *Mycobacterium* species revealed that Rv2038c-41c and UgpAEBC systems are exclusive of pathogenic *Mycobacterium* species and that the LpqY/SugABC system was possibly the first paralog to diverge in the evolution of *M. tuberculosis*. The phylogeny associated with the structural analysis of the *M. tuberculosis* carbohydrate ABC components allowed us to identify that the segregation of the components is mainly based on sequence and structural features related to their functions. Exploring the characteristics and conservation of the interface between permeases and NBDs, we showed that the absence of a NBD in the UspABC system might be compensated by other NBD present in one of the three *M. tuberculosis* carbohydrate ABC transporters.

## Results

### The co-occurrence and similarity of the operons encoding carbohydrate ABC transporters type importer of *Mycobacterium tuberculosis* in different taxa

**Fig. 1:**
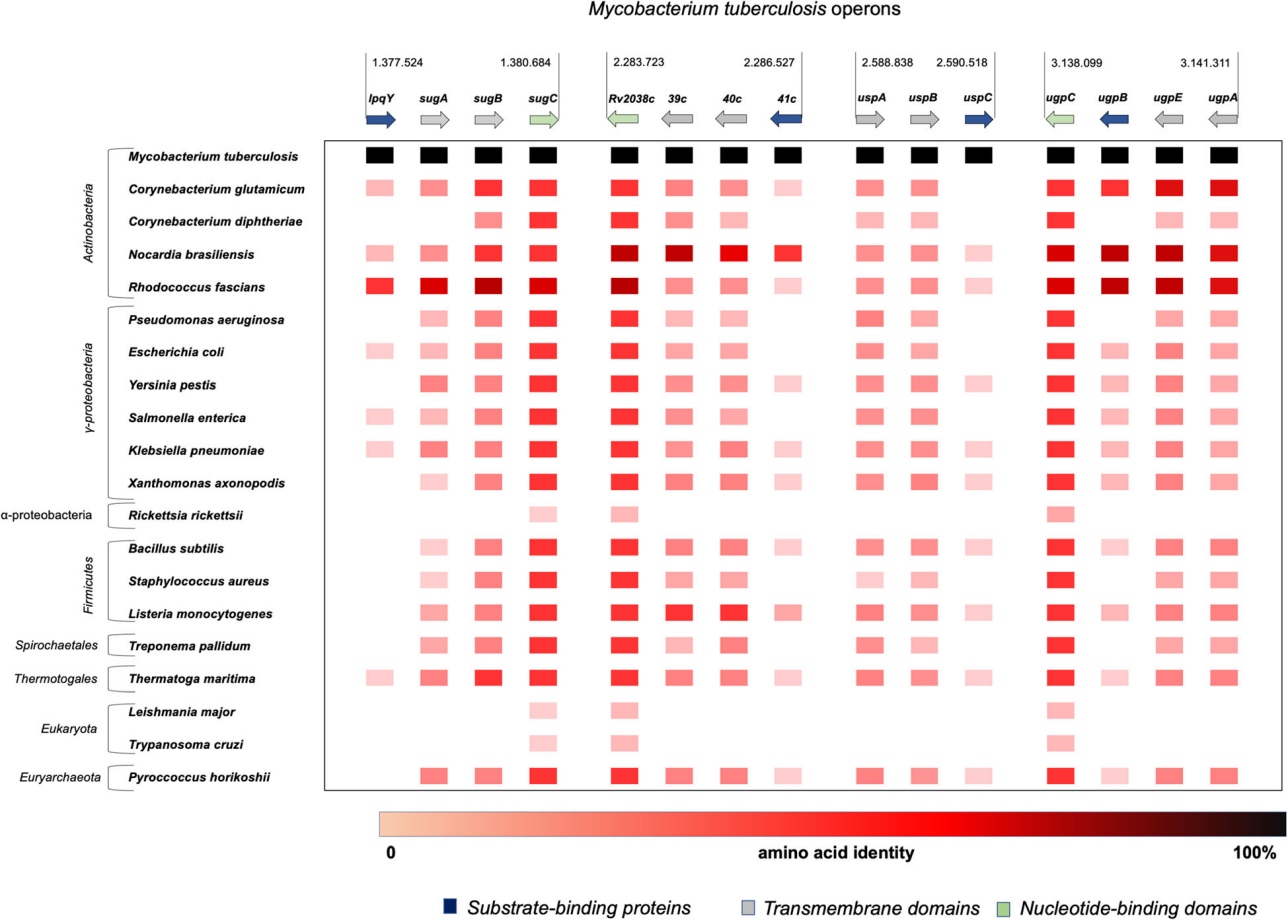
Co-occurrence and genomic proximity of genes encoding for carbohydrate ABC transporters components from *Mycobacterium tuberculosis* across different species. The intensity of red color reflects a conservation level of the component in the species, from the lightest (least conserved protein) to the darkest (most conserved protein). Genes encoding NBD, TMD, and SBP components are shown in green, gray, and blue, respectively. The taxa are shown on the left side

The co-occurrence of components of the four carbohydrate ABC importer of *M. tuberculosis* in different taxa was analysed using String server (Fig. 1). The genes are represented by arrows, whose colors were defined according to their functions. The data show that putative orthologs of the *lpqY/sugABC*, *rv2038c-41c,* and *ugpAEBC* operons are predominantly present in most of the taxa evaluated, except in Eukaryota and *Rickettsia rickettsii* the causative agent of the tick-borne disease named Rocky Mountain spotted fever (RMSF). *uspABC* operon may be misrepresented due to the lack of an evident NDB component, and *ugpCBEA* is highly conserved in the Actinobacteria group. The group of *Corynebacterium diphtheriae* has no conservation of the systems found in *M. tuberculosis*, despite the great relevance of carbohydrates for the metabolism in the genus [24]. *Nocardia brasiliensis*, an actinobacteria that causes pulmonary disease as *M. tuberculosis*, shows conservation of all components related to the Rv2038c-41c and UgpAEBC systems. This bacterium encodes five times more ABC components than *M. tuberculosis* (516 and less than 100, respectively) and in this sense, more resembles a soil bacterium than a pathogenic bacterium [25]. On the other hand, genes related to *lpqY/sugABC* were significantly represented in *Rhodococcus fascians*, a phytopathogenic bacterium, that elicits an accumulation of the disaccharide trehalose during the early stage of plant infection [26]. Similarly, putative orthologs of *lpqY/sugABC* were identified in the oligotrophic bacterium *R. erythropolys* that is able to survive in a completely inorganic medium with no additional carbon source [27].

### Rv2038c-41c and UgpAEBC are restricted to pathogenic species of the *Mycobacterium* genus

In addition to the previous studies, we conducted an analysis of components of putative *M. tuberculosis* carbohydrate ABC importers in 14 different species of *Mycobacterium* genus (Table 2). The choice of species was carefully based on their clinical and biological relevance, including species from well-characterized groups [28]. They consist of human pathogens that belong to the *M. tuberculosis* complex (MTBC) (three *M. tuberculosis* strains, *M. africanum*, and two *M. bovis* strains), pathogenic species non-belonging to the MTBC (*M. avium*, *M. intracellulare*, *M. ulcerans*, *M. marinum*, *M. abscessus,* and *M. leprae*) and the non-pathogenic and environmental species, *M. smegmatis.* The reference list for all of them is presented in Table S1 (Additional file 1). The results revealed that all species of MTBC conserved orthologs of the four ABC transporters studied in this work (amino acid sequence identity of 96 to 100%) and that UgpAEBC is exclusive of MTBC members and *M. marinum*. Although *M. marinum* is not pathogenic for humans, it is responsible for tuberculosis like infections in fishes [29]. We highlight the results for *M. abscessus* (pathogenic but non-belonging to the MTBC) and *M. smegmatis* (non-pathogenic) that lost UgpAEBC and Rv2038-41c transporters. Furthermore, although *M. smegmatis* and other pathogenic species (except *M. leprae*) have a greater number of ABC transporters than MTBC, they do not include the UgpAEBC system (revised in Transport DB 2.0), suggesting this transporter can be related to the uptake of substrates only available in *Mycobacterium* species that cause tuberculosis or tuberculosis like diseases.

**Table 2:**
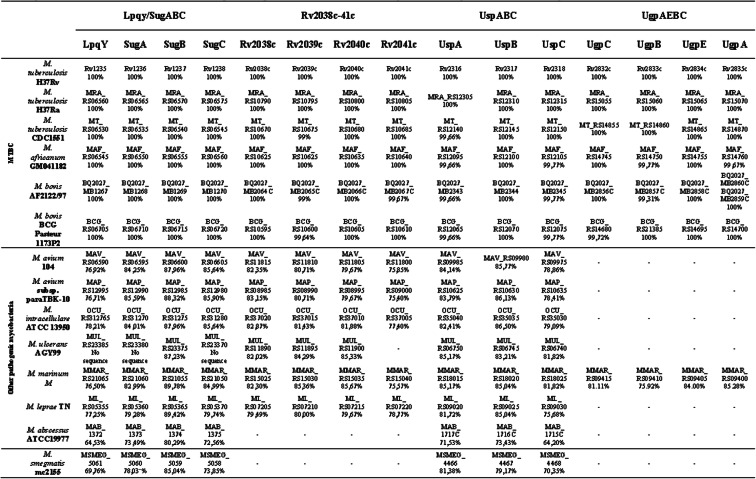
Presence of putative orthologs of the carbohydrate ABC transporters from *Mycobacterium tuberculosis* identified in mycobacterial species. Mycobacteria species are grouped as *M. tuberculosis* Complex (MTBC), other pathogens, and *M. smegmatis*, a non-pathogenic species. The protein sequences were obtained using BLASTp analysis against each strain at NCBI using *M. tuberculosis* H37Rv homologs as the query sequence. The cut-off used was taken using coverage > 90% and amino acid sequence identity > 60%

### Phylogeny and protein variation evidenced in the components of the four *Mycobacterium tuberculosis* carbohydrate ABC transporters

In order to compare the orthologs identified in *Mycobacterium* species, amino acid sequences of all the proteins belonging to the same functional group (NBDs, TMDs, and SBPs) were firstly aligned using Clustal Omega and then submitted to MEGA-X software for phylogenetic analyses. For inferences of phylogeny of conserved proteins, we used the maximum likelihood method [30]. In parallel, we use the available three-dimensional structures or built structural models for all components of the *M. tuberculosis* carbohydrate transporters and use them to map the differences found in the alignments. The models were built using SWISS-MODEL server [31] or Modeller program [32], and the information regarding templates, identities, and model quality details are listed in Table S2 (Additional file 1).

### The nucleotide-binding domains (NBDs)

**Fig. 2:**
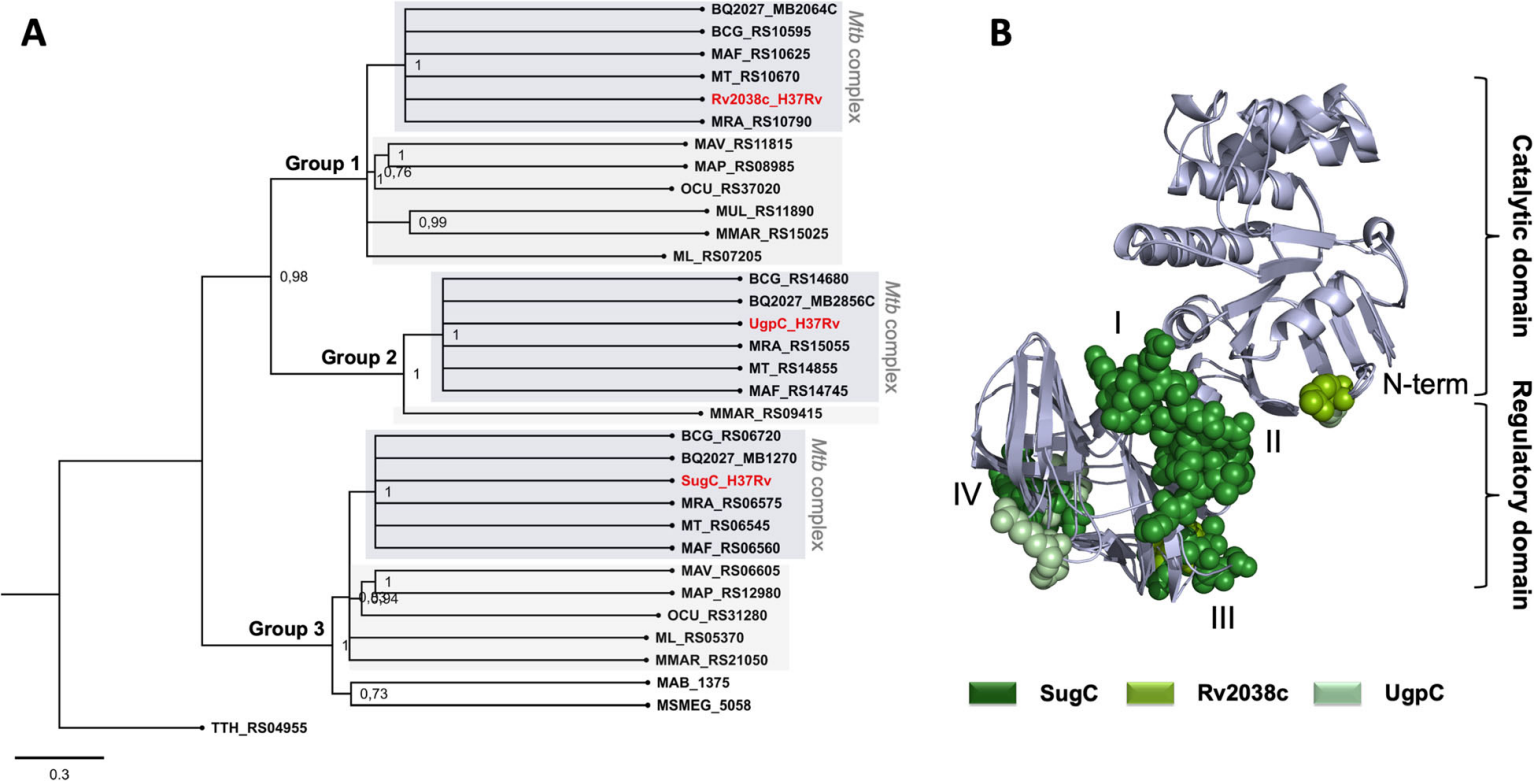
Phylogenetic relationships of NBD components of carbohydrate ABC transporters from species of *Mycobacterium* genus. **A** Phylogenetic tree of *Mycobacterium* carbohydrate NBD components was inferred using a Maximum likelihood method and Jones-Taylor-Thornton (JTT) amino acid substitution model in MEGA-X. Proteins were named with the same NCBI locus tag as presented in Table 1. Sub-groups were colored for better visualization. Dark gray: *M. tuberculosis* complex group (MTBC); light gray: other pathogenic mycobacteria, no color: environmental species*.* The amino acid sequence of TTH_RS04955 from *Thermus thermophilus*, encoding a putative carbohydrate NBD, was used as an outgroup. **B** Structural superposition of the NBDs models showing the regions of each protein that showed differences identified in the amino acid sequence alignment when compared to the others (spheres). The models were built using SWISS-MODEL server [31] and the details of modelling are presented in the Additional file 1 (Table S2). The N-terminal of the proteins is marked but C-terminal is not appearing in the figure

The phylogenetic analysis of NBDs showed that they segregate into three main groups (Groups 1 to 3) each one containing SugC, Rv2038c and UgpC orthologs, respectively (Fig. 2A). Orthologs that were grouped with UgpC (Group 2) belong to the MTBC group and *M. marinum*, and they were closely related to the orthologs of Rv2038c (Group 1). The most distant group of those mentioned above was formed by orthologs from SugC (Group 3). Similarly, the groups 1 and 3 (Rv2038c and SugC NBDs) were divided in two branches, one that consisted of MTBC orthologs, and other that included orthologs of pathogenic mycobacteria and the environmental *M. smegmatis* (Fig. 2A). To assess the differences between the NBDs, we built structural models of the proteins and located the variable regions shown in the alignment after superimposition of them (Fig. 2B, spheres). The *M. tuberculosis* NBDs showed the conserved catalytic domain, similar to the core structure found in many RecA-like motor ATPases [33], and an additional small C-terminal domain that is unique in some ABC transporters, including that related to carbohydrate and ion uptake [34–36]. The amino acid sequence alignment revealed that the catalytic domains were highly conserved but significant differences were found in the regulatory domains, as evidenced by the low sequence identity and presence or absence of specific regions in the proteins (Additional file 2A). The matching and differences between each two proteins is presented in the Additional file 2B. Orthologs from group 3, including SugC, are larger and have at least four (I to IV) additional regions unidentified in the other proteins (Fig. 2B, forest green spheres). It is noted in the alignment that the differences between UgpC and Rv2038 orthologs, which lose the extra SugC regions, are also located in the same region, although the number of differential residues is reduced (Fig. 2B, split pea and pale green spheres, respectively).

### The transmembrane domains (TMDs)

**Fig. 3:**
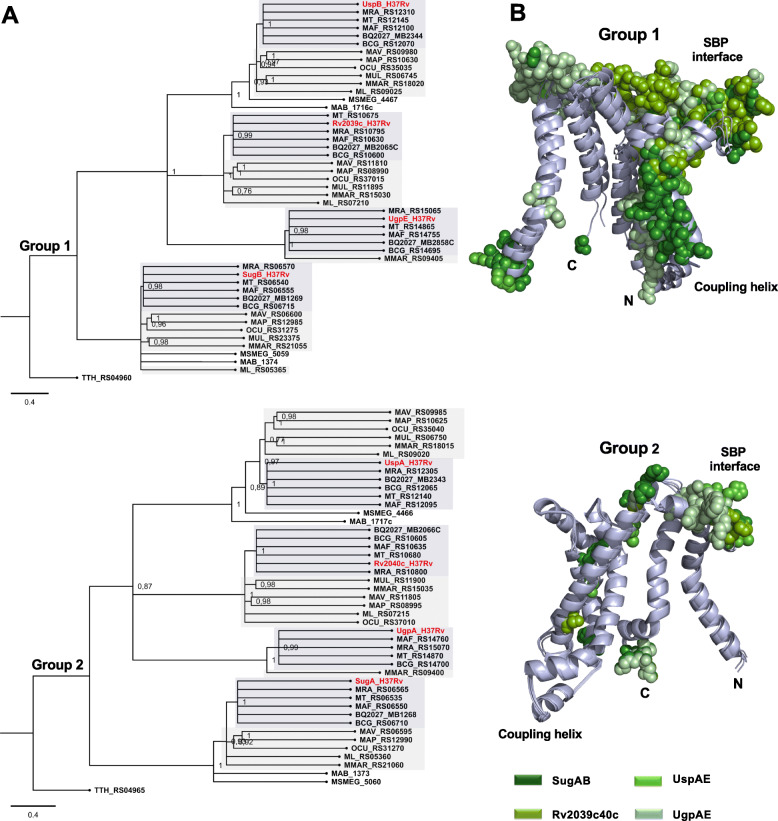
Phylogenetic relationships of TMD components of carbohydrate ABC transporters from species of *Mycobacterium* genus. **A** Phylogenetic tree of *Mycobacterium* carbohydrate TMD components. The tree was inferred using a Maximum likelihood method and Le-Gascuel (LG) amino acid substitution model in MEGA-X. Two trees are showed due to the separation of TMDs in two groups, each one with one member of each transporter. Group 1: SugB, Rv2039c, UspB, and UgpE, and Group 2: SugA, Rv2040c, UspA, and UgpA. Dark gray: *M. tuberculosis* complex group (MTBC), light gray: other pathogenic mycobacteria, and white: environmental species, represented by *M. smegmatis.* The amino acid sequences of TTH_RS04960 and TTH_RS04965 from *Thermus thermophilus*, encoding putative carbohydrate TMDs, were used as outgroup. **B** Structural models of SugB, Rv2039c, UspB, UgpE and SugA, Rv2040c, UspA, and UgpA highlighting the regions that showed significant variation in the amino acid sequences alignments (green colored spheres). Details of the model’s building is shown in the Additional file 1, Table S2

TMD components of ABC importers are responsible for important functions of the transport system, including interaction with the SBPs, formation of the translocation pore through the inner membrane, and interaction with NBDs. The four carbohydrate ABC transporters studied in this work are heterodimers constituted by two different TMDs each. To get phylogenetic insights of the proteins, monomers of each TMDs heterodimer were analyzed separately, forming two groups that we called group 1 and group 2, each one containing one member of each transporter (Additional file 3). Alignments with all amino acid sequences were generated in Clustal Omega for each protein group and used as inputs in MEGA-X software for the building of a rooted tree (Fig. 3A). Group 1 was formed by SugB, Rv2039c, UspB, and UgpE and group 2 by SugA, Rv2040c, UspA, and UgpA. Putative orthologs of SugAB permeases formed a separated group from the three other systems, as shown in SugC NBD group (Fig. 2A). According to the alignments, proteins from group 1 have 5 to 30 additional residues than those from group 2 and seem to be the most variable component in the architecture of *M. tuberculosis* carbohydrate ABC transporters, once alignments by pair revealed large insertions/deletions (indels) regions (Additional file 3A). To identify the location of possible variation regions in the proteins, a structural model of each *M. tuberculosis* TMD component was built (Additional file 1, Table S2), and proteins were compared by pairs (Additional file 3B). In general, the main differences among the proteins are located in the N-terminal that faces the NBDs and in the loop between helices TM1 and TM2, which in all models consists of a region that is more exposed to the periplasm and might be accessed by the SBP. Main differences were found in group 1 of TMDs, mostly including helices 3 and 4. (Fig. 3B, green spheres). No differences were identified in the coupling helices (Additional file 3C) as well as in the helices that form the pore. The SugA orthologs conserved the most residues that in *M. smegmatis* LpqY-SugABC transporter interact with trehalose, but differently, *M. smegmatis* SugB His118 is not conserved (Additional file 3A, in red).

### The substrate-binding proteins (SBPs)

**Fig. 4:**
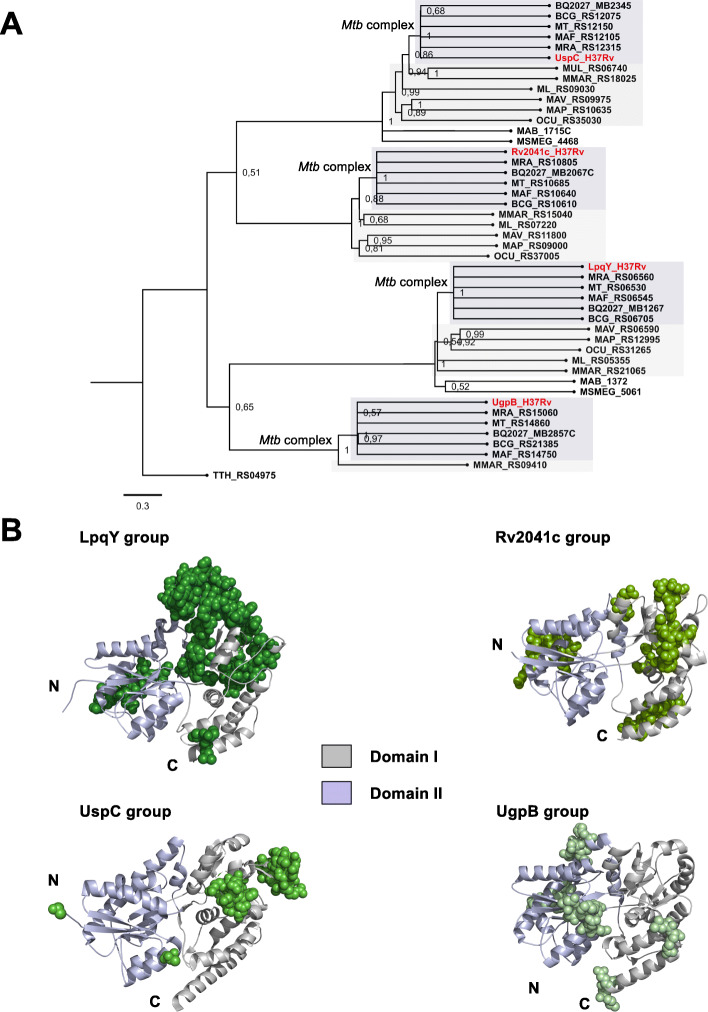
Phylogenetic relationships of SBP components of carbohydrate ABC transporters from species of *Mycobacterium* genus. **A** Phylogenetic tree of *Mycobacterium* carbohydrate SBP components. The tree was inferred using a Maximum likelihood method and Whelan and Goldman (WAG) amino acid substitution model in MEGA-X. Dark gray: *M. tuberculosis* complex group (MTBC), light gray: other pathogenic mycobacteria, no color: environmental species*.* The amino acid sequence of TTH_RS04975 from *Thermus thermophilus* was used as an outgroup. **B** Crystallographic structures of UspC (PDB code: 5K2X) and UgpB (PDB code: 4MFI), and structural models of LpqY and Rv2041c were used for representation of regions that showed variation. Regions of amino acid insertion/deletion are represented in colored spheres

The role of SBPs in ABC importers is of great relevance since they are the components responsible for the affinity and specificity of the transport systems. They perform the substrate uptake and transference to the TMDs for the translocation. The interaction between SBPs and TMDs triggers structural movements that will result in the change of resting to an active state of the transporter [37]. The phylogenetic tree built with the amino acid sequences alignment of carbohydrate SBPs from *Mycobacterium* species showed that each component separated in a unique group with their orthologs (Fig. 4A). UgpB orthologs, exclusive of MTBC and *M. marinum,* are closer to the orthologs of LpqY than UspC and Rv2041c. The available structures of *M. tuberculosis* UgpB (PDB code: 4MFI) [20], UspC (PDB code: 5K2X) [19], and the structural models of LqpY and Rv2041c (Additional file 1, Table S2) were used for mapping the differences evidenced in the amino acid sequence alignments (Fig. 4B). Structurally, the *M. tuberculosis* carbohydrate SBPs consist of two globular domains, N-terminal (domain I) and C-terminal (domain II) that are connected by a hinge, in which interface it is located the substrate-binding site. A comparison among the protein groups in the alignment (Additional file 4) allowed us to identify specific regions with amino acids indels, as shown in Fig. 4B. UspC is the shortest protein and, differently from the three others, it has a deletion of two sets of amino acids, respectively in domain I (opposite to the entrance of binding pocket) and domain II (Fig. 4B, yellow spheres). LpqY, Rv2041c, and UgpB show amino acid insertions in domains I and II (Fig. 4B, spheres). These regions are not involved in the carbohydrate-binding site but can indirectly affect the structure of the binding-pockets. UgpB is the protein that shows more sites of variability, in both domains, including regions that directly affect the substrate-binding pocket (Fig. 4B, Additional file 4).

### Substrate-binding pocket comparison of the *M. tuberculosis* H37Rv SBPs

**Fig. 5:**
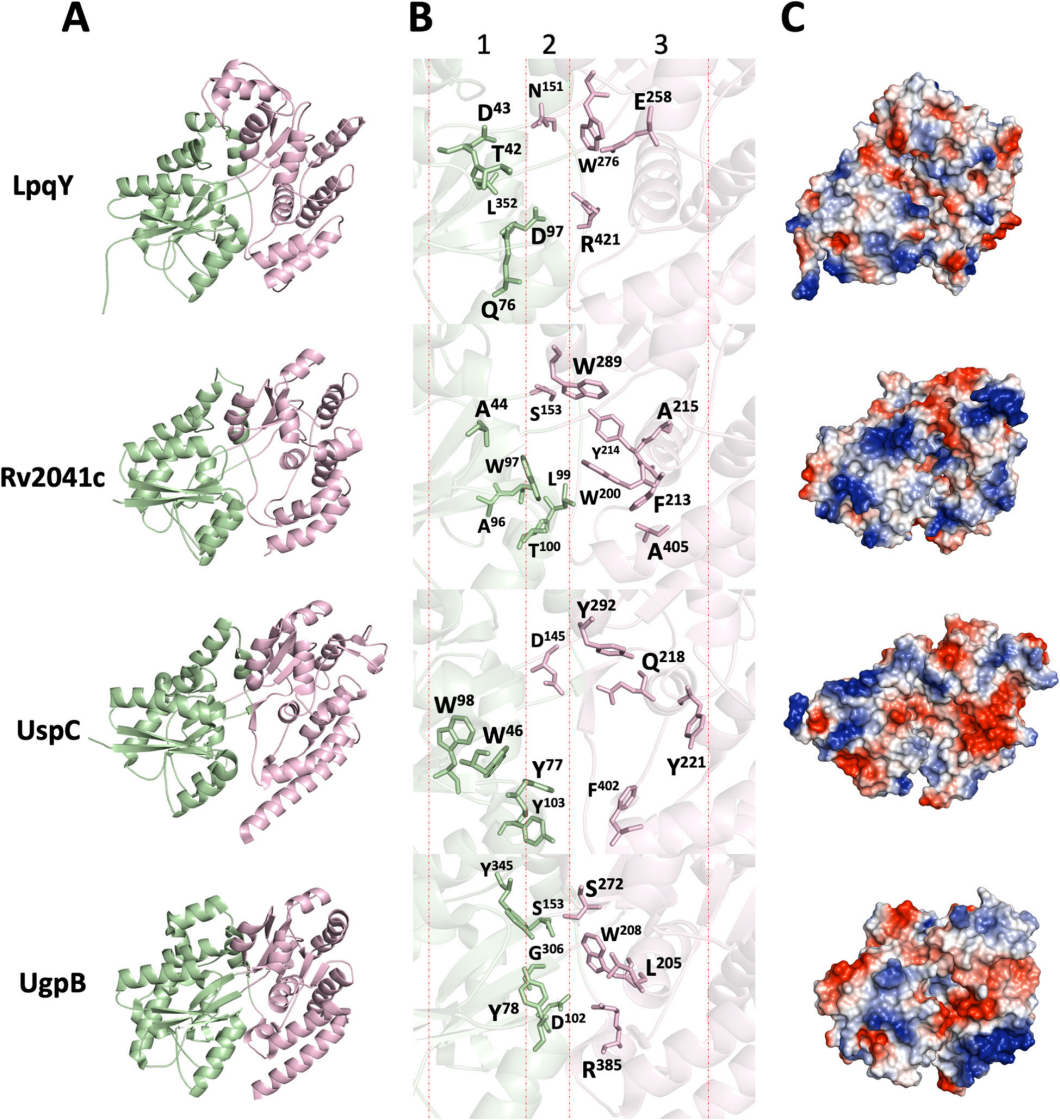
Comparison among the four carbohydrate-binding proteins from *M. tuberculosis.* **A** The crystallographic structures of UgpB (PDB ID: 4MFI) and UspC (PDB ID: 5K2X) were compared to the structural models of Rv2041c and LpqY. Proteins are shown as cartoons with the domain I (N-terminal) and II (C-terminal) colored in green and pink, respectively. **B** Mapping of the residues that form the substrate-binding pockets according to the crystallographic structures and structural models. **C** Electrostatic potential of the proteins from the pocket entrance perspective. Blue: positive, red: negative, gray: neutral

The previous phylogenetics analysis showed that the four *M. tuberculosis* SBP segregate in different groups. We compared the available three-dimensional structures of the *M. tuberculosis* UspC and UgpB and the molecular models of LpqY and Rv2041c. The general structure consisted of two alpha-beta domains (or lobes), characteristic of periplasmic-binding proteins, where domain I is smaller than domain II and it is more conserved (Fig. 5A). The structures conserved the three-dimensional folding, but clearly revealed substrate-binding pockets with significant differences, which is in accordance with phylogenetic analyses that separate them in four groups (Fig. 5B, Additional file 5A). *Mtb* LpqY model was based on the *M. smegmatis* LpqY three-dimensional structure bound to trehalose (PDB 7CAF_E) that shared 69% of amino acid sequence identity (94% coverage). The comparison of model and structure revealed a ligand-binding pocket very similar. From six residues that interact with the sugar in *M. smegmatis* protein [18], *M. tuberculosis* LpqY conserves four (Asp97, Asn151, Glu288 and Arg421). Residues Asn41 and Glu42 of *M. smegmatis* LpqY are replaced by Asp 41 and Thr42 in the *M. tuberculosis* protein, strongly suggesting it also can bind trehalose. LpqY folding is also closely related to the trehalose-binding protein of *T. litoralis* (TMBP, PDB code: 1EU8) [34] and the maltose-binding protein of *T. maritima* (MalE3, PDB code: 6DTQ) [38]. The structural model of mature protein Rv2041c was generated by SWISS-MODEL server [31] based on the structural similarity with *Listeria monocytogenes* Lmo0181 (PDB code: 5F7V) [39] (Additional file 1, Table S2). However, Rv2041c did not conserve the pocket residues and apparently, there is no possibility for interaction with cycloalternan, the substrate of Lmo0181. The SBPs superimposed well and revealed three subsites (Fig. 5B). Subsites 1 and 3 are rich in aromatic residues, and subsite 2 shows hydrophilic residues, also conserved in the alignment (Additional file 5A). Similar to what we observed in the ligand pockets, the electrostatic potential of the proteins revealed differences in the entrance of the pocket and TMDs interface regions (Fig. 5C), in accordance with the phylogenetic analysis and supporting the variability of substrates. The structural alignment of the proteins revealed five conserved regions, four of them probably involved in the structural folding (Additional file 5B).

### Characterization of the interaction between TMD coupling helices and NBDs

**Fig. 6:**
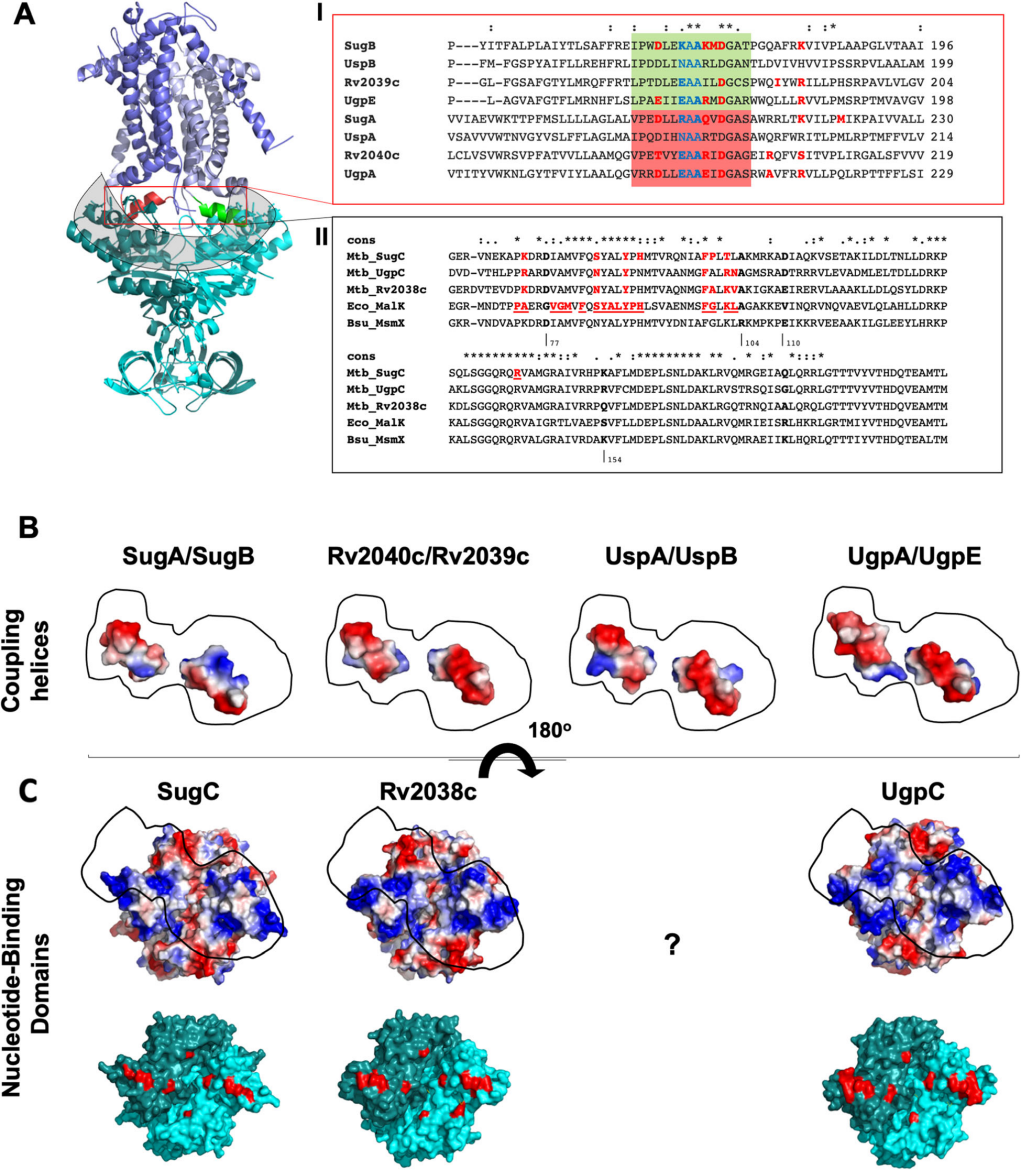
The interface between coupling helices and NBDs of *Mycobacterium tuberculosis* carbohydrate ABC transporters*.* **A** Structural model of a carbohydrate transporter showing the dimers of TMDs (pale blue and blue) and NBDs (cyan and deep cyan). The two coupling helices from each monomer are colored in green and red, respectively, and the NBD region that faces the helices is highlighted in a grey box. (I) and (II) Local amino acid sequence alignments of TMDs and NBDs, respectively. Coupling helices are colored as in the structural model. Sequences of the *E. coli* MalK and *B. subtilis* MsmX NBDs were included in the alignment for comparison. Residues of the interface between MalK and MalF/G are shown in red underlined and those that determine promiscuity in MsmX are in bold. **B** Electrostatic potential of TMDs coupling helices and NBDs shown as surface. The area in black line highlights the position of the interaction. Blue: positive, red: negative, grey: neutral. **C** Structure of the NBD dimers in surface showing the two monomers and the regions (area in black line) that might interact with coupling helices. The NBD structures are also shown in cyan surface with the putative residues important in the interaction in red

TMDs in ABC transporters are responsible for the pore formation and they play an essential role in the activation of the NBDs during the transport, mediating hydrophobic and hydrophilic interactions with the energy domains to stabilize the complex in the different states of activity. The absence of a NBD in UspABC arised the questioning if could exist some promiscuity among NBDs of the aforementioned *M. tuberculosis* transporters. Incomplete ABC transporters missing ATPase domains were identified in many genomes of different species as well as orphan ATPase domains [40]. Multitask NBDs, such as *Bacillus subtilis* MsmX, capable to energize more than one transporter seem to be common in the CUT1 subfamily (di-, tri- and oligosaccharides) [40, 41]. In *E. coli,* MalK and UgpC NBDs were functionally changeable maintaining the functions of the transporters but not the regulatory function [42]. To evaluate this possibility, we firstly compared in the TMDs, the characteristics of the predicted coupling helices, which were localized between TM4 and TM5 (Fig. 6A, green and red helices, and Additional file 3). It was observed that the coupling helices show variation in the first residue of the EAA motif [(E/N/R/K)AA]. UspA/B have an asparagine (non-polar), Rv2039/40c and UgpA/E glutamic acid, and SugA/B lysine or arginine (positively charged), indicating differences in the electrostatic potential of the helices (Fig. 6A-I, in blue). Using GREMLIN server analysis for complexes [43], we predicted the residues performing interactions between TMD and NBD (Additional file 1, Table S3). In relation to TMD residues, it was evident that the first and third residues of the EAA motif play an important role, as well as a set of charged residues, including a highly conserved aspartate (Fig. 6A-I). These residues essentially perform interactions with similar residues in the NBDs. To support our analysis, the putative coupling helices/NBD interfaces were mapped in the structural models (Fig. 6A, gray region) and had their amino acid sequences aligned. The amino acid sequences of *E. coli* MalK and *B. subtilis* MsmX, whose interactions with TMDs were largely explored, were included in the alignment. The MalK residues that interact with MalF and MalG were underlined in Fig. 6A-II, including Phe81 to Tyr87 that accommodate the MalF and MalG helices. The comparison of this region with *M. tuberculosis* sequences reveals high conservation of residues that form an hydrophobic environment in the models. Although the GREMLIN analyses did not identify as many residues as in MalK, the identified residues from *M. tuberculosis* proteins were structurally aligned with MalK. We also looked at the conservation of residues of *M. tuberculosis* NBDs in comparison with *B. subtilis* MsmX. Residues Asp77, Arg104, Glu110 and Lys154 are conserved in NBDs that can complement MsmX activity but they are replaced by hydrophobic residues in MalK [44] (Fig. 6A-II, in black bold). Similarly to MsmX, *M. tuberculosis* proteins conserve an aspartate in the equivalent position of Asp77 and residues with negative and positive charges such as Glu110 and Lys154, respectively. The only change in *M. tuberculosis* proteins was a replacement of Arg104 by an alanine, as observed in *E. coli* MalK. Not directly interacting with the coupling helices, MsmX Arg104 is responsible for adding charge to the environment and it is supposed to maintain the needed structure of the region for transport [44]. Rv2038c and UgpC proteins seem to share more residues in common than with UspC (Fig. 6A-II). The electrostatic potential of the *M. tuberculosis* coupling helices and NBDs was evaluated and revealed a complementary interface consisting of prominent negative charges in the helices (Fig. 6B), and a set of 8 bunches of positive charges that are spread along the interface generated by the NBD dimers (Fig. 6C).

## Discussion

The cellular and molecular mechanisms involved in *M. tuberculosis* nutrition have been largely studied and despite many uncertainties, the importance of carbohydrates and lipids has arised. ABC transporters have a clear contribution during colonization of the host environment, through nutrient scavenging, and evasion or resistance to host defenses. *M. tuberculosis* has four ABC transporters dedicated to carbohydrate transport, which studies have demonstrated they play important role in recycling of trehalose, transport of amino sugars and glycerolphosphocholine, but also in biofilm formation, virulence and immunogenicity [12, 19–22]. In this work, we made a comparative analysis of these transporters detaching the phylogenetic, structural and structural aspects of their components. In general, the analysis of co-occurrence of *M. tuberculosis* carbohydrate ABC transporter components reveals they are poorly represented with sequences that show low similarity. Actinobacteria is the unique group in which these components are more representative, mainly LpqY-SugABC, Rv2038-41c and UgpAEBC systems. There is a consensus for the presence of NBDs, reflecting the high level of conservation of these proteins in ABC transporters. In addition, there is a sporadic absence of periplasmic components and some of the TMDs, which is partly due to their low conservation of the amino acid sequence. In the case of permeases, it is possible that the absence of a component is indicative of the formation of homodimers of TMDs, as evidenced in other ABC systems. Interestingly, the complete absence of these carbohydrate ABC components was evidenced in the *Rickettsia rickettsia* and Eukariota group, which was represented by the two human parasites *Leichmania major* and *Trypanosoma cruzi*. *R. rickettsia* is a strict intracellular pathogen associated with arthropods [45] that has a reduced genome. The identified ABC transporters present in this microorganism are mainly dedicated to heme acquisition, lipids, toluene tolerance and drug extrusion suggesting that carbohydrates don’t represent a significant source of nutrients. The distribution of the four ABC transporters in representative species inside the *Mycobacterium* genus revealed they conserved at least orthologs of two ABC transporters, LpqY/SugABC and UspABC. Interestingly, was the genomic comparison between *M. smegmatis* and *M. tuberculosis. M. smegmatis* is a fast-growing non pathogenic species that can transport via ABC transporter systems, a variety of sugars: β-glucosides such as chitobiose, α-galactosides (melibiose), β-xylosides (xylobiose), xylose, arabinose, and sugar alcohols. Differently, *M. tuberculosis* has a reduced number of ABC transporters dedicated to carbohydrate uptake, and from the available data until the moment, both species do not seem to share substrates. These differences reflect the lifestyles of *M. smegmatis* and *M. tuberculosis* in their natural habitats, the soil, and human body, respectively [13]. Additionally, our results additionally showed that Rv2038c-41c and UgpAEBC are not only restricted to *M. tuberculosis* species, but also distributed in other pathogenic species of *Mycobacterium* genus. The presence of the UgpAEBC system exclusively in species of *M. tuberculosis* complex group and *M. marinum* reveal the relevance of this system for the bacilli. UgpB, the SBP component, is a substrate of twin-arginine translocation (Tat) pathway, conserved in different bacterial species [20, 21], upregulated during infection, and essential for virulence and survival in several pathogens [46, 47]. Although *M. marinum* has fish as its official host, it is also capable of infecting humans causing cutaneous tuberculosis. The conservation of UgpAEBC transporter in this species could be important for carbohydrate and phosphate sources uptake during the infection in humans [20, 21]. Indeed, NMR studies of the metabolomic profiling of intact lung tissue at various stages of *M. tuberculosis* infection have revealed a significant increasing of the UgpAEBC substrate (GPC) during the early stages of infection [48]. Rv2038-41c, the closest system to UgpEABC, is also conserved only in pathogenic species suggesting an importance in the infection and pathogenesis processes. Indeed, although the substrate of this transporter is not clear, the SBP Rv2041c was related to intracellular adaptation within the host and considered relevant for the pathogen biology and virulence, since it was upregulated in phagosome acidic and hypoxic conditions [22].

Based on the amino acid sequence alignments and evaluation of structural parameters of the protein models, we observed that the components from the four transporters keep clustering in four groups, separated by functional and structural differences. Evidence for that is highlighted in the differences found in the regulatory domains from NBDs, in the substrate-binding pockets from periplasmic-binding proteins, and in the interface of TMDs. All NBDs analyzed conserve a catalytic subdomain, while the main differences are observed in the C-terminal regulatory region. Despite that NBDs mostly segregate according to sequence differences in a region that lies between Walker A and B motifs and includes the structurally more diverse helical subdomain, [49], our phylogenetic analyses showed that the differences on the regulatory domains of *M. tuberculosis* NBDs play an essential role in their segregation. The regulatory subdomain of several carbohydrate NBDs conserved short sequence motifs such as FVAxFIGSP, GψRPE, and ExxG, (ψ is an apolar residue and x is any amino acid) and C-terminal glycine and phenylalanine residues that can serve as signatures [50]. These motifs and residues are present in *E. coli* MalK and UgpC, *Thermococcus litoralis* MalK, and others, and also in *M. tuberculosis* SugC, Rv2038c, and UgpC. SugC presents a larger regulatory domain and previous phylogenetic analysis demonstrated that all the mycobacterial SugC proteins cluster together depicting the high sequence conservation. Interestingly, these proteins branched out together with homologous proteins from *Pseudomonas syringae* and *Klebsiella pneumoniae,* which are plant and animal pathogens, respectively [27].

The analysis of the TMDs showed predominant differences in the interfaces with SBPs and NBDs, especially that from group 2, indicating their relevance in the recognition and transport mechanism. Our analysis suggests that *M. tuberculosis* carbohydrate TMDs could have diverged from an ancient SugB in group B, and SugA in group A. Comparative studies of TMDs from ABC importers show they conserve six transmembrane segments (TMs) that could be originated from a duplication of a primordial protein containing 3 TMs [51]. The electrostatic potential of the pores formed by the TMDs in the four systems is mainly apolar with positive charges at the entrance and end of the pore, emphasizing the similar characteristics of the substrates.

The periplasmic components showed the highest diversity indicating they select different sets of substrates. On the contrary of NBD and TMD components, the phylogenetic analysis resulting from SBPs showed a different pattern of divergence. It could be explained by the quite discussed diversity of SBP components. The structural comparisons show that the diversity regions could not be necessarily associated with the binding site, but additional regions of the proteins that play distinct roles in the transport system and the bacterium physiology. The structural similarities, the evidence of solvent-accessible aromatic side chains in the binding cleft, and the characteristic acidic molecular surface corroborate their role as carbohydrate transporters. Previous works have proposed the ability of *M. tuberculosis* to exchange carbohydrates and lipids as energy sources, an essential feature for *M. tuberculosis* survival in macrophages [9, 52]. Carbon and phosphate can be acquired by the action of bacillus phospholipases and glycerol phosphodiesterases on the host’s phospholipids. The proximity of the periplasmic proteins to the amino-sugars of the cell wall could facilitate the acquisition of substrates like GPC, trehalose, and chitobiose by UgpB, UspC and LpqY respectively [15, 19, 20].

Structural details shared by all SBPs components of carbohydrate ABC transporters were analyzed and they exhibited a topology of subcluster DI in the structural classification of substrate-binding proteins [53]. Although the structure of Rv2041c is based on a model, the apparent topology and its molecular weights above 40 kDa, suggest it belongs to the same subcluster. Despite apparent conservation in the structure of these proteins, the electrostatic potential of the ligand-binding cleft does not reflect this conservation, indicating they have preferences and affinities for different substrates. Finally, in an attempt to evaluate the putative promiscuity of the *M. tuberculosis* carbohydrate NBDs, our study compared amino acids and charges in the coupling helices and NBDs of the carbohydrate transporters of *M. tuberculosis.* The sequences of the *B. subtilis* MsmX, a multitask NBD, and *E. coli* MalK, were included in the alignment for comparison. The results of this analysis showed the conservation of residues that form the hydrophobic cleft in NBDs for interaction with the coupling helices (Phe81-Tyr87, in *E. coli* MalK). Still, it was possible to notice that from the four residues of *B. subtilis* MsmX (Asp77, Arg104, Glu110 and Lys154) evaluated as signature of multitask NBDs [44], the *M. tuberculosis* proteins conserved the aspartate in position 77 in all the sequences, and Glu110 in Rv2038c, but replaced by an aspartate in SugC and UgpC. Although not identical, the residues in these key positions contribute to the more charged environment of this region when compared to MalK. The presence of charged residues in the vicinity of the coupling helices promotes conformational changes and greater plasticity in this region, favoring a greater number of interactions. Thus, the set of results from this comparison suggests that the carbohydrate NBDs could alternate between transporters in *M. tuberculosis*. This observation, however, depends on further studies to be confirmed.

## Conclusions

The results showed that the segregation of the components of the *M. tuberculosis* carbohydrate transporters was determined by sequential alterations that culminated in structural changes, important for determination of their specificity and function. Still, significant differences were found in the regulatory domains of the NBDs and in the ligand-binding pockets of the SBPs, both defining the function of the transporters. The conservation of charges and residues suggests that there is promiscuity among the NBDs of the analyzed ABC transporters. All similarities and differences addressed in this work can serve as a basis for further studies of development of inhibitors, using mainly the transporters Rv2038-41c and UgpAEBC as targets, since they were conserved in pathogenic species and are not identified in eukaryotes.

## Methods

### Searching for orthologs

The genes coding carbohydrate ABC transporters components of *M. tuberculosis* were obtained from Mycobrowser (https://mycobrowser.epfl.ch/) [54] or KEGG (https://www.genome.jp/kegg/) [55] servers. The co-occurrence analysis across different taxa was evaluated using String server (https://string-db.org/) [56]. The comparative genomic analysis across *Mycobacterium* species was described by Machowski and collaborators [57]. Briefly, we choose 15 reference genome sequences of *Mycobacterium* strains deposited in GenBank (https://www.ncbi.nlm.nih.gov/genbank/) (Additional file 1, Table S1), and searched for homologs of the carbohydrate ABC transporter components of *M. tuberculosis*. Identified amino acid sequences were used for BLASTp (Basic Local Alignment Search Tool**,** https://blast.ncbi.nlm.nih.gov/Blast.cgi) at NCBI (https://www.ncbi.nlm.nih.gov/). Amino acid sequence alignments and identity of each sequence related to *M. tuberculosis* ortholog were performed using Clustal Omega (https://www.ebi.ac.uk/Tools/msa/clustalo/) [58].

### Phylogenetic analysis

The alignments and phylogenetic analysis of ABC transporter components (NBDs, TMDs, or SBPs) were visualized and performed using MEGA-X [59]. The alignments were edited manually, using visual inspection of possible conserved and non-conserved regions. Models with the lowest BIC scores (Bayesian Information Criterion) were considered to describe the best substitution pattern. For each model, AICc value (Akaike Information Criterion, corrected), Maximum Likelihood value (*lnL*), and the number of parameters (including branch lengths) were calculated. The phylogeny was established from analysis of Maximum Likelihood. As an external group, the components of a putative carbohydrate ABC transporter from *Thermus thermophilus* (NCBI locus tag: TTH_RS04955–70/TTH_RS04975) were used. The robustness of the inferred trees was tested by bootstrap analyses (500 replicates). All trees generated were visualized using FigTree (http://tree.bio.ed.ac.uk/software/figtree/).

### Construction of the structural models and interactions

Molecular models of the ABC transporter components were generated using SWISS-MODEL server [31] or Modeller program [32], which used structural coordinates of ortholog proteins deposited in the Protein Data Bank (PDB), as described in detail in Table S2 (Additional file 1). All models were subsequently validated for their stereochemical quality using the program MolProbity [60]. The final model to be used for further analysis was chosen based on the geometrical parameters. All figures were generated using the program PyMOL (The PyMOL Molecular Graphics System, Version 1.2r3pre, Schrödinger, LLC) [61].

### SBP characterization and binding site prediction

Signal peptide sequences of the substrate-binding proteins were predicted using SignalP-5.0 Server [62]. Rv2041c substrate-binding site was predicted after the structural superimposition of the three *M. tuberculosis* carbohydrate-binding proteins with the Rv2041c model (Additional file 1, Table S3). The pocket volumes of the proteins were calculated using the program CASTp [63] with a default probe radius of 1.4 A and MetaPocket [64].

### Characterization of the TMDs and prediction of interfaces with NBDs

The validation of the TMD models was performed in conjunction with the prediction of transmembrane helices using the programs TMHMM Server v. 2.0 [65] and TOPCONS [73] and analysis of the position, residue composition (EAA conservation) and predicted structure of the coupling helices. The Complexes program from Gremlin server (http://gremlin.bakerlab.org/) [43], was used for prediction of the interface between TMDs (coupling helices) and NBDs, based on evolutionary information. Since the region of interaction was larger than 60 residues, we used the e-value threshold of E-06 and number of iterations with Jackhammer to 4. We accepted interprotein residue pairs with a scaled score ≥ 1.30 and a probability > 0.88 as co-varying pairs (evolutionary couplings, ECs).

## Supplementary Information

**Additional file 1: Table S1.** List of *Mycobacterium* species used in this work. **Table S2.** Proteins used as templates for structural modelling of the carbohydrate ABC transporter components of *M. tuberculosis* H37Rv*.* SBP: substrate-binding protein; TMD: transmembrane domain; NBD: nucleotide-binding domain. I-TASSER server or Modeller program were used for structural modeling. **Table S3.** Paired alignment of carbohydrates NBDs and TMDs from *M. tuberculosis* H37Rv. The analysis was performed using Gremlin complexes**.** NBD: nucleotide-binding domain, TMD: transmembrane domain. Scaled score: “normalized coupling strength”, a coupling strength larger than one indicates higher than average coupling between two residues. Probability: P (contact | scaled_score, seq/len). I_probability: P (contact | scaled_score, seq/len, top_inter_score).

**Additional file 2. **Structural and amino acid sequence differences found in the *M. tuberculosis* carbohydrate NBD components. A Amino acid sequence alignment of NBDs. The alignment made with Clustal Omega only shows the regulatory domains where the main differences are observed. B Structural comparison of NBDs and the variable positions identified in the amino acid alignment. Differences between every two proteins can be evidenced by the colored spheres. The structural models show regions of amino acid insertion/deletion identified when two proteins are compared. The percentage in each box represents the amino acid sequence identity between two orthologues. Structural models of Rv2038c, UgpC, and SugC were built as described in Additional file 1, Table S2.

**Additional file 3. **Sequential and structural differences found in the *M. tuberculosis* H37Rv carbohydrate ABC transporters TMDs. A Amino acid sequence alignment of TMDs produced with the Clustal Omega program. Proteins can be divided in group 1 (SugB, UspB, UgpB and Rv2039c) and group 2 (SugA, UspA, UgpA and Rv2040c, coloured in gray). The EAA residues of the coupling helix are shown in yellow. Residues forming the interface between SugA and SugB that interact with trehalose in the structure of *M. smegmatis* LpqY-SugABC transporter are shown in red. *M. tuberculosis* SugB residues in cyan are the corresponding in *M. smegmatis* SugB forming the scoop loop. B Structural comparison of TMDs. Comparison can be made for each two models and the coloured residues represent the position of variable regions between two proteins. The percentage in each box represents the amino acid sequence identity between the two proteins. Models of all proteins were built using the SWISS-MODEL program (see Additional file 1, Table S2). C Prediction of topology of the TMDs components from *M. tuberculosis* H37Rv carbohydrate ABC transporters. Amino acid sequences of the proteins were submitted to the TOPCONS program. The red bars highlight the position of coupling helices.

**Additional file 4. **Sequential and structural position of the main differences between the *M. tuberculosis* sugar transporters SBPs. **A** Amino acid sequence alignment of the SBPs showing the four groups (bold letters). The alignment was made using Clustal Omega. Residues in red are involved in the ligand binding. **B** Proteins in cartoon represent the modelled structures of all SBPs, except for UgpB and UspC that have crystallographic structures (PDB IDs: 4MFI and 5K2X, respectively). After the amino acid sequence alignment of each two structures different regions were shown by coloured spheres and can be compared. Models of LpqY and Rv2041c were built using the SWISS-MODEL program [31] (see Additional file 1, Table S2).

**Additional file 5. **Amino acid sequence alignment of the *M. tuberculosis* H37Rv sugar binding proteins and structural information. A Alignment of UgpB, Rv2041c, LpqY and UspC obtained with the Expresso program in the T-coffee server (http://tcoffee.crg.cat/apps/tcoffee/do:expresso). Amino acids that form the ligand-binding pocket are highlighted in green. The region of the three subsites is shown. B Three-dimensional structure of UgpB in cartoon showing the five regions with highest conservation (left). The putative function of each region is represented in the structure in color surface (right).

## Data Availability

All data generated or analysed during this study are included in this published article and its additional files. Genomic sequences of *Mycobacterium* species were obtained from the National Center for Biotechnology Information – NCBI (https://www.ncbi.nlm.nih.gov/). All NCBI accession numbers were listed in the Additional file 1, Table S1. All *Mycobacterium spp.* protein sequences were obtained from Mycobrowser (https://mycobrowser.epfl.ch/). Sequences of other microorganisms were obtained in the Kyoto Encyclopedia of Genes and Genomes - KEGG (https://www.genome.jp/kegg/).

## Abbreviations

ABC: ATP-Binding Cassette
MTBC: *Mycobacterium tuberculosis* Complex
NBD: Nucleotide-Binding domain
PDB: Protein Data Bank
SBP: Substrate-Binding Protein
TM: Transmembrane
TMD: Transmembrane domain
