# Supplementary material for: Comparison of carbohydrate ABC importers from Mycobacterium tuberculosis
Source: BMC Genomics. 2021 Nov 19;22:841. doi: 10.1186/s12864-021-07972-w (PMC8603345; doi:10.1186/s12864-021-07972-w)
Supplement: Supplementary file 1 [file 12864_2021_7972_MOESM1_ESM.docx]

Structural and phylogenetic insights of ABC transporters catalysing carbohydrate uptake from *Mycobacterium tuberculosis*

Lilia I. De la Torre, José G. Vergara Meza, Sindy Cabarca Barreto, André G. Costa-Martins, Andrea Balan

Additional File 1

Table S1. List of *Mycobacterium* species used in this work.

Mycobacterium species/strain	NCBI accession number	Reference link
M. tuberculosis H37Rv	NC_000962	http://www.ncbi.nlm.nih.gov/pubmed/20980199
M. tuberculosis H37Ra	NC_009525	http://www.ncbi.nlm.nih.gov/pubmed/18584054
M. tuberculosis CDC1551	NC_002755	http://www.ncbi.nlm.nih.gov/pubmed/12218036
M. africanum GM041182	NC_015758	http://www.ncbi.nlm.nih.gov/pubmed/22389744
M. bovis AF2122/97	NC_002945	http://www.ncbi.nlm.nih.gov/pubmed/12788972
M. bovis sp. BCG Pasteur 1173P2	NC_008769	http://www.ncbi.nlm.nih.gov/pubmed/17372194
M. avium 104	NC_008595	Direct Submission
M. avium sub. *paratuberculosis* K-10	NC_002944	http://www.ncbi.nlm.nih.gov/pubmed/16116077
M. intracellulare ATCC 13950	NC_016946	http://www.ncbi.nlm.nih.gov/pubmed/22535933
M. ulcerans AGY99	NC_008611	http://www.ncbi.nlm.nih.gov/pubmed/17210928
M. marinum M	NC_010612	http://www.ncbi.nlm.nih.gov/pubmed/18403782
M. abscessus ATCC19977	NC_010397	http://www.ncbi.nlm.nih.gov/pubmed/23804391
M. smegmatis mc²155	NC_008596	Direct Submission
M. leprae TN	NC_002677	http://www.ncbi.nlm.nih.gov/pubmed/11234002

Table S2. Characteristics of proteins used as templates for structural modelling of the carbohydrate ABC transporter components of *M. tuberculosis* H37Rv and parameters for evaluation of the model quality. SBP: substrate-binding protein; TMD: transmembrane domain; NBD: nucleotide-binding domain. The construction of the models was carried out with the SWISS-MODEL server [37].

Target Proteins	Template Information				Model Quality
M. tuberculosis ABC transporter components	Microorganism/ Protein function	Query cover/ Identity (%)	PDB_chain	Resolution (Å)	QMEAN
LpqY Rv1235 (SBP)	*Mycobacterium smegmatis* LpqY	99/69	7CAE_E	3.44	-3.17
SugA Rv1236 (TMD)	*Mycobacterium smegmatis* SugA - TMD	99/79	7CAF_D	3.30	-3.81
SugB Rv1237 (TMD)	*Mycobacterium smegmatis* SugB - TMD	100/85	7CAD_D	3.41	-4.81
SugC Rv1238 (NBD)	*Mycobacterium smegmatis* SugC - NBD	98/74	7CAD_A	3.41	-3.46
Rv2038c (TMD)	*Pyrococcus horikoshii* ATP-binding cassette of multisugar transporter	97/49	1V43_A	2.20	-1.82
Rv2039c (TMD)	*Mycobacterium smegmatis* SugB - TMD	94/29	7CAD_D	3.41	-6.05
Rv2040c (NBD)	*Mycobacterium smegmatis* SugA - TMD	92/24	7CAF_D	3.30	-6.42
Rv2041c (SBP)	*Geobacillus stearothermophilus* AbnE - SBP	90/25	6RKJ_A	1.60	-3.12
UspA Rv2316 (TMD)	*Mycobacterium smegmatis* SugB - TMD	88/17	7CAD_D	3.41	-7.18
UspB Rv2317 (TMD)	*Mycobacterium smegmatis* SugB - TMD	97/28	7CAD_D	3.41	-4.59
UspC Rv2318 (SBP)	*Mycobacterium tuberculosis* UspC - SBP	100/100	5K2X	1.50	-
UgpC Rv2832c (NBD)	*Pyrococcus horikoshii* ATP-binding cassette of multisugar transporter	97/50	1V43_A	2.20	-2.15

UgpB<br>Rv2833c<br>(SBP)	*Mycobacterium tuberculosis*<br>UgpB - SBP	100/100	4MFI<br>6R1B	1.50<br>2.27	-
UgpE<br>Rv2834c<br>(TMD)	*Mycobacterium smegmatis*<br>SugB - TMD	97/22	7CAD_D	3.41	-4.98
UgpA<br>Rv2835c<br>(TMD)	*Mycobacterium smegmatis*<br>SugA - TMD	95/26	7CAF_D	3.30	-5.05

Table S3. Paired alignment of carbohydrates NBDs and TMDs from *M. tuberculosis*. The analysis was performed using Gremlin complexes. NBD: nucleotide-binding domain, TMD: transmembrane domain. Scaled score: "normalized coupling strength", a coupling strength larger than one indicates higher than average coupling between two residues. Probability: P (contact | scaled_score, seq/len). I_Prob (I Probability): P (contact | scaled_score, seq/len, top_inter_score).

NBD	TMD	Scaled Score	Probability	I_Prob
		**SugC x SugA**		
48_N	200_R	3.27	1.00	1.00
99_F	215_K	3.24	1.00	1.00
102_T	215_K	2.38	1.00	1.00
99_F	202_A	1.77	1.00	0.97
88_Y	205_D	1.68	1.00	0.96
84_S	197_D	1.65	1.00	0.96
140_R	197_D	1.45	0.99	0.92
90_H	220_M	1.44	0.99	0.92
74_K	203_Q	1.38	0.99	0.90
100_P	205_D	1.38	0.99	0.89
		**SugC x SugB**		
48_N	166_K	3.24	1.00	1.00
99_F	181_K	3.16	1.00	1.00
99_F	168_A	2.57	1.00	1.00
102_T	181_K	2.25	1.00	0.99
88_Y	171_D	1.90	1.00	0.98
84_S	163_D	1.89	1.00	0.98
74_K	169_K	1.51	1.00	0.94
53_L	170_M	1.36	0.99	0.89
		**Rv2038c x Rv2039c**		
49_R	174_E	3.17	1.00	1.00
100_F	189_R	2.94	1.00	1.00
100_F	176_A	2.35	1.00	1.00
103_K	189_R	2.32	1.00	1.00
89_Y	179_D	1.84	1.00	0.98
85_N	171_D	1.82	1.00	0.98
75_K	177_I	1.41	0.99	0.91
		**Rv2038c x Rv2040c**		
49_R	189_E	3.62	1.00	1.00

100_F	204_S	3.46	1.00	1.00
100_F	191_A	2.35	1.00	1.00
103_K	204_S	2.33	1.00	1.00
85_N	186_T	1.90	1.00	0.98
89_Y	194_D	1.78	1.00	0.98
101_A	194_D	1.63	1.00	0.96
104_V	200_R	1.49	1.00	0.93
75_K	192_R	1.48	1.00	0.93
		**UgpC x UgpA**		
100_F	214_R	3.50	1.00	1.00
49_R	199_E	3.41	1.00	1.00
100_F	201_A	2.32	1.00	1.00
103_R	214_R	2.25	1.00	0.99
85_N	196_D	1.70	1.00	0.97
89_Y	204_D	1.63	1.00	0.96
101_A	204_D	1.51	1.00	0.94
104_N	210_A	1.49	1.00	0.93
75_R	202_E	1.44	0.99	0.92
		**UgpC x UgpE**		
49_R	168_E	3.23	1.00	1.00
100_F	183_R	3.06	1.00	1.00
100_F	170_A	2.35	1.00	1.00
103_R	183_R	2.32	1.00	1.00
89_Y	173_D	1.89	1.00	0.98
85_N	165_E	1.85	1.00	0.98
75_R	171_R	1.44	1.00	0.91
101_A	173_D	1.36	0.99	0.89
