# Supplementary material for: Comparison of carbohydrate ABC importers from Mycobacterium tuberculosis
Source: BMC Genomics. 2021 Nov 19;22:841. doi: 10.1186/s12864-021-07972-w (PMC8603345; doi:10.1186/s12864-021-07972-w)
Supplement: Supplementary file 2 [file 12864_2021_7972_MOESM2_ESM.docx]

Structural and phylogenetic insights of ABC transporters catalysing carbohydrate uptake from *Mycobacterium tuberculosis*

Lilia I. De la Torre, José G. Vergara Meza, Sindy Cabarca Barreto, André G. Costa-Martins, Andrea Balan

Additional File 2

A

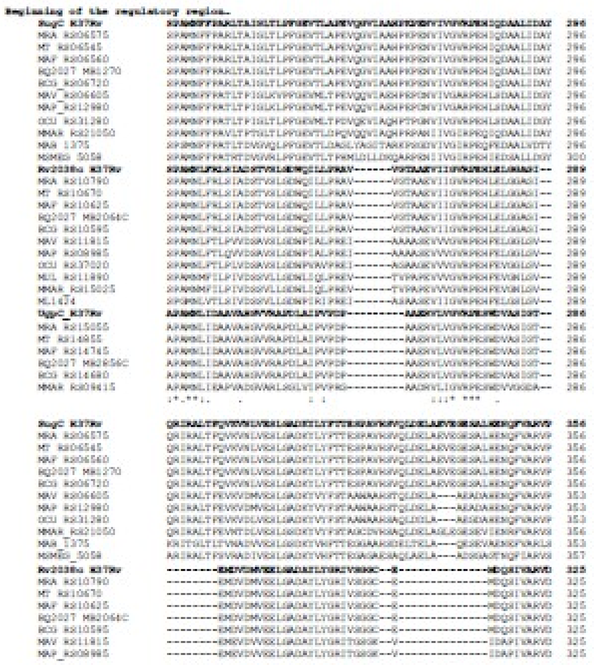

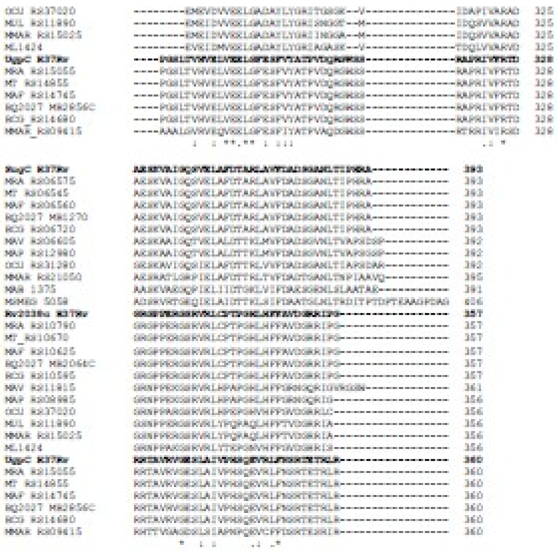

B

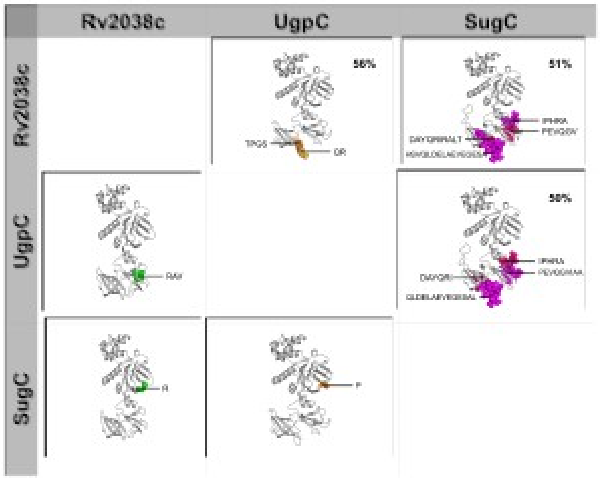

Sequential and structural differences found in the *M. tuberculosis* H37Rv NBDs of carbohydrate ABC transporters. A Amino acid sequence alignment of NBDs. The alignment made with Clustal Omega only shows the sequence corresponding to the regulatory domains where the main differences were observed. B Localization of the differences evidenced after comparison of two proteins at a time in their three-dimensional models. Proteins are presented in grey cartoon with coloured spheres representing the localization of differences in the structures. The percentage in each box represent the amino acid sequence identity between two proteins. Models of Rv2038c, UgpC and SugC were built in SWISS-MODEL [37] (see Additional file 1, Table S2).
