# Supplementary material for: Comparison of carbohydrate ABC importers from Mycobacterium tuberculosis
Source: BMC Genomics. 2021 Nov 19;22:841. doi: 10.1186/s12864-021-07972-w (PMC8603345; doi:10.1186/s12864-021-07972-w)
Supplement: Supplementary file 3 [file 12864_2021_7972_MOESM3_ESM.docx]

Structural and phylogenetic insights of ABC transporters catalysing carbohydrate uptake from *Mycobacterium tuberculosis*

Lilia I. De la Torre, José G. Vergara Meza, Sindy Cabarca Barreto, André G. Costa-Martins, Andrea Balan

Additional File 3

A

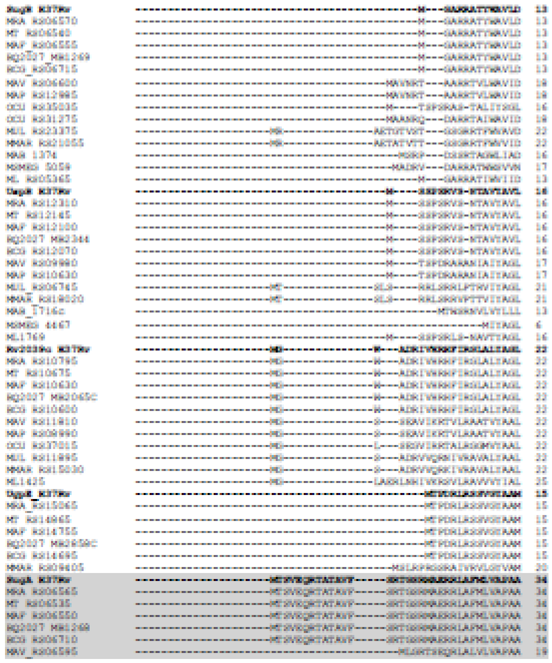

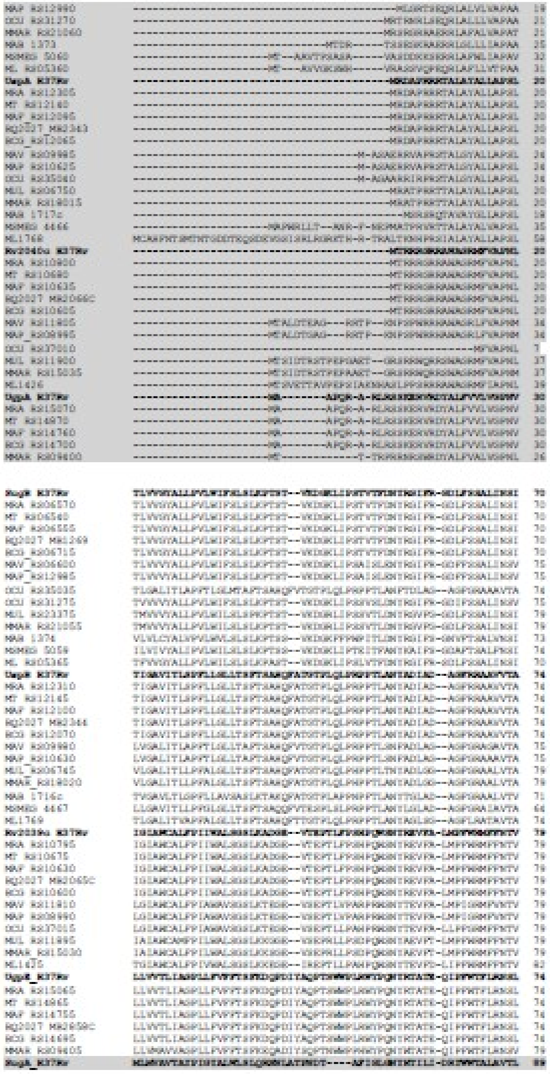

```
MKA RS06565       MLMSAVTATPIGTALALSLQRNNLATPNDT----AFIGLGNTHTILI-DRTWNTALAVTL  89
MT RS06535        MLMSAVTATPIGTALALSLQRNNLATPNDT----AFIGLGNTHTILI-DRTWNTALAVTL  89
MAF RS06550       MLMSAVTATPIGTALALSLQRNNLATPNDT----AFIGLGNTHTILI-DRTWNTALAVTL  89
BQ2027 MB1269     MLMSAVTATPIGTALALSLQRNNLATPNDT----AFIGLGNTHTILI-DRTWNTALAVTL  89
BCG RS06710       MLMSAVTATPIGTALALSLQRNNLATPNDT----AFIGLGNTHTILI-DRTWNTALAVTL  89
MAV RS06595       ILMLAVTATPIGTAVALSLQRNNLAAPNDT----AFVSLGNTATILA-DRTWNTALAVTL  76
MAP RS12990       ILMLAVTATPIGTAVALSLQRNNLAAPNDT----AFVSLGNTATILA-DRTWNTALAVTL  76
OCU RS31270       LLMLTVTATPIGTAVALSLQRNNLAVPDDT----AFIGLGNTVTILT-DRTWNTALAVTL  76
MMAR RS21060      ILMLLVIGTPIGTAMALSLQRNNLATPNKT----AFIGLGNTQTILV-DRTWNTALAVTA  76
MAB 1373          LLMLAVTATPICTAIALSVQKTSPASPQDR----KFVWFGNTITVLA-DRTWNAALSVTL  80
MSMEG 5060        LLMLAVTATPIGTAVALSLQRTNLAKPNDT----AFIGLANTVTVLT-DRTWNTAFAVTL  87
ML RS05360        MLMLVVTATPIGTAVALSLQRTSLATPNKT----VFIGLGNTQTILT-DFTWNTALAVTL  86
UgpA H37Rv        VVVVAAPLLLPILVVVMLSLAHHDLLGPL------HTVGLTNWRSVLT-DSGFADSLVVTA  73
MKA RS12305       VVVVAAPLLLPILVVVMLSLAHHDLLGPL------HTVGLTNWRSVLT-DSGFADSLVVTA  73
MT RS12140        VVVVAAPLLLPILVVVMLSLAHHDLLGPL------HTVGLTNWRSVLT-DSGFADSLVVTA  73
MAF RS12095       VVVVAAPLLLPILVVVMLSLAHHDLLGPL------HTVGLTNWRSVLT-DSGFADSLVVTA  73
BQ2027 MB2343     VVVVAAPLLLPILVVVMLSLAHHDLLGPL------HTVGLTNWRSVLT-DSGFADSLVVTA  73
BCG RS12065       VVVVAAPLLLPILVVVMLSLAHHDLLGPL------HTVGLTNWRSVLT-DSGFADSLVVTA  73
MAV RS09985       PGVLAAPLLLPILVVIMLSLCHHDLLGPL------RFVGLSNWRSVLT-DASFGNSLMVTA  77
MAP RS10625       PGVLAAPLLLPILVVIMLSLCHHDLLGPL------RFVGLSNWRSVLT-DASFGNSLMVTA  77
OCU RS35080       PGVLAAPLLLPILVVIMLSLCHHDLLGPL------RFVGLSNWRSVLT-DGTFGNSLIVTA  77
MUL RS06750       PGVLAAPLLLPILVVIMLSLYHHDLLGPL------HTVGLANWRSVLA-DSDFGNSLVVTA  73
MMAR RS18015      PGVLAAPLLLPILVVIMLSLYHHDLLGPL------HTVGLANWRSVLA-DSDFGNSLVVTA  73
MAB 1717c         PGVAAAPLLLPILVVVMLSLCHHDLLGPI------KFVGLDNWRSVLT-DGTFGNSLLVTL  71
MSMEG 4666        PGVVTPPLLLPMLVVVMLSLAHHDLLGPI------KFVGLDNWTSVLT-DPAFGNSLVVTL  88
ML1768            PGVVTPPLLLPIIVVIMLSLPHHDLLGPL------HTVGLANWRSVLT-DPNFANSLIVTA  111
Rv2040c H37Rv     AAVVVVPMLFPLGFSLYMSFQKWDLFTAA------TFVRLDNFRKLFTDDPLFLIAVVNTA  76
MKA_RS10800       AAVVVVPMLFPLGFSLYMSFQKWDLFTAA------TFVRLDNFRKLFTDDPLFLIAVVNTA  76
MT RS10680        AAVVVVPMLFPLGFSLYMSFQKWDLFTAA------TFVRLDNFRKLFTDDPLFLIAVVNTA  76
MAF RS10635       AAVVVVPMLFPLGFSLYMSFQKWDLFTAA------TFVRLDNFRKLFTDDPLFLIAVVNTA  76
BQ2027 MB2066C    AAVVVVPMLFPLGFSLYMSFQKWDLFTAA------TFVRLDNFRKLFTDDPLFLIAVVNTA  76
BCG RS10605       AAVVVVPMLFPLGFSLYMSFQKWDLFTAA------TFVRLDNFRKLFTDDPLFLIAVVNTA  76
MAV RS11905       VAVAVPMLFPLGFSLYMSFQRWDVPTPP------KFVGLKNPTDLFSDDPLFLIAIRNTV  88
MAP RS09995       VAVAVPMLFPLGFSLYMSFQRWDVPTPP------KFVGLKNPTDLFSDDPLFLIAIRNTV  88
OCU RS37010       VAVAVPMLFPLGFSLYMSFQRWDVPTPP------KFVGLKNPRKLFTADPLFLIAIRNTV  61
MUL RS11900       AAVAVPLLFPLGFSLYMSFQRWDLPAAP------TFVGLQNFRKLFTDDPLFLIALANSV  91
MMAR RS15035      AAVAVPLLFPLGFSLYMSFQRWDLPAAP------TFVGLQNFRKLFTDDPLFLIALANSV  91
ML1426            AAVAVPMLFPLGFSLYMSFQKWDMPTPP------VFVGLANFQKLFTDDPLFLIALCNSV  93
UgpA H37Rv        ALLLLPVTRPLADNIRLSFFDWRVSDPS-A----SFVGLGNTTENFT-RSDTRQIVFNTA  84
MKA RS15070       ALLLLPVTRPLADNIRLSFFDWRVSDPS-A----RFVGLGNTTENFT-RSDTRQIVFNTA  84
MT RS14870        ALLLLPVTRPLADNIRLSFFDWRVSDPS-A----RFVGLGNTTENFT-RSDTRQIVFNTA  84
MAF RS14760       ALLLLPVTRPLADNIRLSFFDWRVSDPS-A----RFVGLGNTTENFT-RSDTRQIVFNTA  84
BCG RS14700       ALLLLPVTRPLADNIRLSFFDWRVSDPS-A----RFVGLGNTTENFT-RSDTRQIVFNTA  84
MMAR RS09400      ALLLLPVTRPLVDNIRLSFFDWRISDPT-A----SFVGFGNTIENFS-RPDTRQIVANTA  80
                           *.  .  :                  *:                   :
                                                           #
SugB H37Rv        GIGLITTTVIAVVLGAMAAYAVARLEFPGRRLLIGAALLIT-MPVXISLVTPLFNIERAIG  129
MKA RS06570       GIGLITTTVIAVVLGAMAAYAVARLEFPGRRLLIGAALLIT-MPVXISLVTPLFNIERAIG  129
MT RS06540        GIGLITTTVIAVVLGAMAAYAVARLEFPGRRLLIGAALLIT-MPVXISLVTPLFNIERAIG  129
MAF RS06555       GIGLITTTVIAVVLGAMAAYAVARLEFPGRRLLIGAALLIT-MPVXISLVTPLFNIERAIG  129
BQ2027 MB1269     GIGLITTTVIAVVLGAMAAYAVARLEFPGRRLLIGAALLIT-MPVXISLVTPLFNIERAIG  129
BCG RS06715       GIGLITTTVIAVVLGAMAAYAVARLEFPGRRLLIGAALLIT-MPVXISLVTPLFNIERAIG  129
MAV RS06600       GIGLITTAVAVLLGAMAAYAVARLDFPGRRLLIGATLLIT-MPPAISLVTPLFNIERFLG  134
MAP RS12985       GIGLITTAVAVLLGAMAAYAVARLDFPGRRLLIGATLLIT-MPPAISLVTPLFNIERFLG  134
OCU RS35035       LMTAVILVGQLTPSVLAAYAFARLQFPGRDALFWVYIATL-MVPATVTIVPMYIMMAQLG  133
OCU RS31275       GIGLITTAIAVTLGAMASYAIARLAFPGRRALVGATLLIT-MPPAISLVTPLFNIERFVG  134
MUL RS23375       GIGLITTTVIAVLLGAMAACAVARLDFPGRRLLVGVTLLIT-MPPAISLVTPLFNIERRIG  138
MMAR RS21055      GIGLITTTVIAVLLGAMAAYAVARLDFPGRRLLVGVALLIT-MPPAISLVTPLFNIERRIG  138
MAB 1374          GIGLIATVIAVSVGTMAAYAVARLDFPGRRALIGAALLIA-MPPQISLVTPIPNIERSVG  132
MSMEG 5059        GIGLITTIIAVVIGGMAAYAVARLQFPGRQLLIGVALLIA-MPPMISLVTPIFNMMRSIG  133
ML RS05365        GIGLITTTVIAVMPGAMAAYAIARLAFPGRRALLVGTTLLVT-MPPAISLVTPLFNIERRVG  129
UgpE H37Rv        LMTAVILLGQLTPSVLAAYAFARLQFPGRDALFWVYVATL-MVPGTVTVVPLYIMMAQLG  133
MKA RS12310       LMTAVILLGQLTPSVLAAYAFARLQFPGRDALFWVYVATL-MVPGTVTVVPLYIMMAQLG  133
MT RS12145        LMTAVILLGQLTPSVLAAYAFARLQFPGRDALFWVYVATL-MVPGTVTVVPLYIMMAQLG  133
MAF RS12100       LMTAVILLGQLTPSVLAAYAFARLQFPGRDALFWVYVATL-MVPGTVTVVPLYIMMAQLG  133
BQ2027 MB2344     LMTAVILLGQLTPSVLAAYAFARLQFPGRDALFWVYVATL-MVPGTVTVVPLYIMMAQLG  133
BCG RS12070       LMTAVILLGQLTPSVLAAYAFARLQFPGRDALFWVYVATL-MVPGTVTVVPLYIMMAQLG  133
MAV RS09980       LMTAVILVGQLTPSVLASYAFARLAFPGRDALFWVYIATL-MVPGTVTIVPMYIMMAQLG  134
MAP RS10630       LMTAVILVGQLTPSVLASYAFARLAFPGRDALFWVYIATL-MVPGTVTIVPMYIMMAQLG  134
MUL RS06745       LMTTVILLGQMTPSVLAAYAFARLAFPGRDGLFWVYIATL-MVPATVTVVPLYIMMAQLG  138
MMAR RS18020      LMTAVILLGQMTPSVLAAYAFARLAFPGRDGLFWVYIATL-MVPATVTVVPLYIMMAQLG  138
MAB 1716c         LMTAIIVIGQLTPSVLAAYAFARLAFPGRDGLFWIYLATL-MIPATVTVVPMYIMLTQVG  130
MSMEG 4467        LMTAVILLGQLVPSVLAAYAFARLAFPGRDVLFWVYLATL-MVPATVTVVPLYLIMAKAG  123
ML1769            LMTAVILLGQLTPSVLASYAFARLAFPGRDTLFWAYIMTL-MVPATVTVVPMYMMMAQVG  133
Rv2039c H37Rv     LYASCVTASQVFFCSLASYAFARLQFPGRDTLFVLYLSTL-MVPLTVTVIPQVILMRIVG  138
MKA RS10795       LYASCVTASQVFFCSLASYAFARLQFPGRDTLFVLYLSTL-MVPLTVTVIPQVILMRIVG  138
MT RS10675        LYASCVTASQVFFCSLASYAFARLQFPGRDTLFVLYLSTL-MVPLTVTVIPQFILMRIVG  138
MAF RS10630       LYASCVTASQVFFCSLASYAFARLQFPGRDTLFVLYLSTL-MVPLTVTVIPQFILMRIVG  138
BQ2027 MB2065C    LYASCVTASQVFFCSLASYAFARLQFPGRDTLFVLYLSTL-MVPLTVTVIPQFILMRIVG  138
BCG RS10600       LYASCVTASQVFFCSLASYAFARLQFPGRDTLFVLYLSTL-MVPLTVTVIPQFILMRIVG  138
MAV RS11810       LYASCVTASHVFFCSLASYAFARLDFPGRNTLFVVYLSTL-MVPLTVTVIPQFLIMRTLG  138
MAP RS08990       LYASCVTASHVFFCSLASYAFARLDFPGRNTLFVVYLSTL-MVPLTVTVIPQFLIMRTLG  138
OCU RS37015       LYASCVTASHVFFCSLASYAFARLDFPGRDALFVVYLSTL-MVPLTVTVIPQFLVMRTLG  138
MUL RS11895       LYASCVTASQIFFCSLASYAFARLAFPGRDTLFVLYLSTL-MVPLTVTVIPHFILMRTVG  138
MMAR RS15030      LYASCVTASQIFFCSLASYAFARLAFPGRDTLFVLYLSTL-MVPLTVTVIPQFILMRTVG  138
ML1425            LYASYVTVSQVFFCSLASYAFARLQFPGRDALFVLYLSTL-MVPLTVTIIPQFILMRVLG  141
UgpE H37Rv        IITSVLAVVKPTLSVLAAPSLVFVKFPGRTAVFLVIIAAL-MVPNQSTVISNYALISKLG  133
MKA_RS15065       IITSVLAVVKPTLSVLAAPSLVFVKFPGRTAVFLVIIAAL-MVPNQSTVISNYALISKLG  133
```

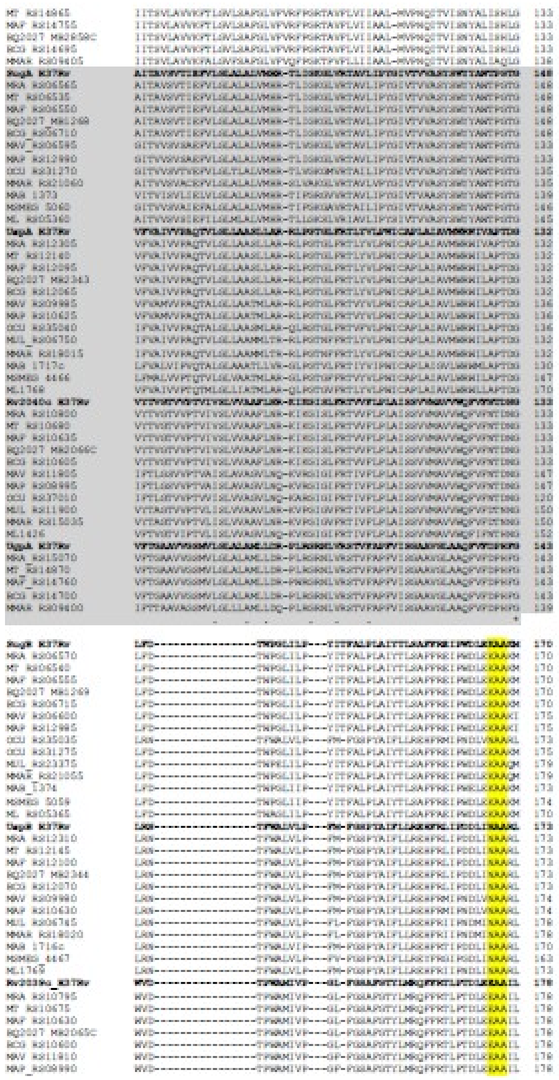

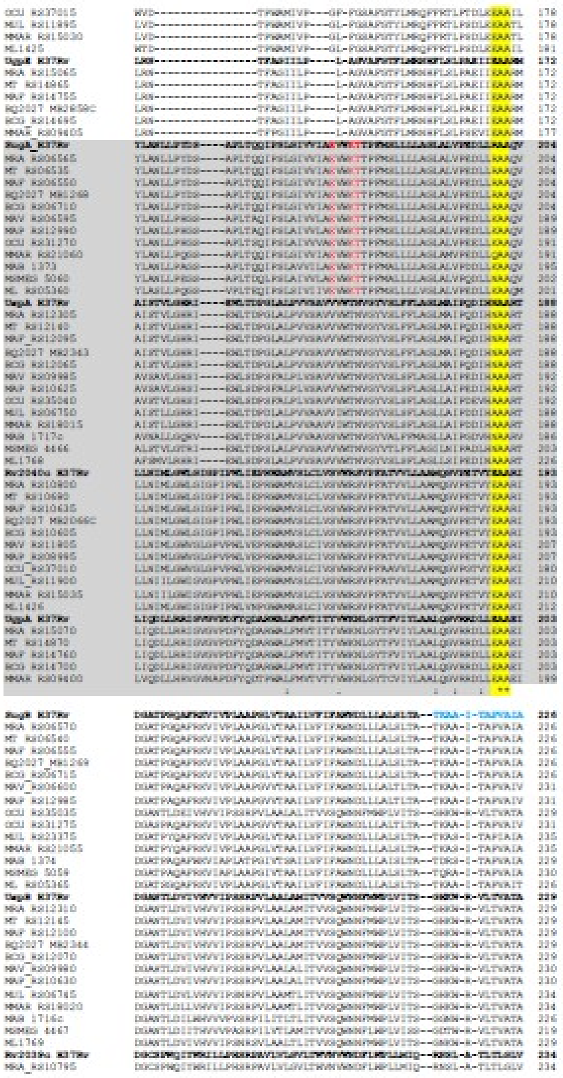

```
MT RS10675         DGCSPWQITWRILLPHSKPAVLVLSVLTWVNVWNDFLWPLLMIQ--RNSL-A-TLTLGLV  234
MAF RS10630        DGCSPWQITWRILLPHSKPAVLVLSVLTWVNVWNDFLWPLLMIQ--RNSL-A-TLTLGLV  234
BQ2027 MB2065C     DGCSPWQITWRILLPHSKPAVLVLSVLTWVNVWNDFLWPLLMIQ--RNSL-A-TLTLGLV  234
BCG RS10600        DGCSPWQITWRILLPHSKPAVLVLSVLTWVNVWNDFLWPLLMIQ--RNSL-A-TLTLGLV  234
MAV RS11810        DGCTPWQVTWRILLPHAKPAVMVLAVLTWVNVWNDFLWPLLMIQ--KSSL-A-TLTLGLV  234
MAP RS08990        DGCTPWQVTWRILLPHAKPAVMVLAVLTWVNVWNDFLWPLLMIQ--KSSL-A-TLTLGLV  234
OCU RS37015        DGCSPWQVTWRILLPHAKPAVMVLAVLTWVNVWNDFLWPLLMIQ--RNSL-A-TLTLGLV  234
MUL RS11895        DGCSPWQITWRILLPHAKPAVMVLSVLTWVNVWNDFLWPLLMVQ--KDSI-A-TLTLGLV  234
MMAR RS15030       DGCSPWQITWRVLLPHAKPAVMVLAVLTWVNVWNDFLWPLLMVQ--KDSI-A-TLTLGLV  234
ML1425             DGCSPWQITWRVLLPHAKPAVGVLAVLTWVNVWNDFLWPLLMIQ--RNSL-A-TLTLGLV  237
UgpE_H37Rv         DGARWMQLLLAVVLPMSRPTMVAVGVITVVNEWNKYLWPFLMSD--DRSV-A-PLPIGLT  228
MRA_RS15065        DGARWMQLLLAVVLPMSRPTMVAVGVITVVNEWNKYLWPFLMSD--DRSV-A-PLPIGLT  228
MT RS14965         DGARWMQLLLAVVLPMSRPTMVAVGVITVVNEWNKYLWPFLMSD--DRSV-A-PLPIGLT  228
MAF RS14755        DGARWMQLLLAVVLPMSRPTMVAVGVITVVNEWNKYLWPFLMSD--DRSV-A-PLPIGLT  228
BQ2027 MB2859C     DGARWMQLLLAVVLPMSRPTMVAVGVITVVNEWNKYLWPFLMSD--DRSV-A-PLPIGLT  228
BCG RS14695        DGARWMQLLLAVVLPMSRPTMVAVGVITVVNEWNKYLWPFLMSD--DRSV-A-PLPIGLT  228
MMAR RS09405       DGARWMQLLPAVVLPMSGPTMVAPGIITVVNEWNKYLWPFLMSD--DRSV-A-PLPVGLT  233
SugA H37Rv         DGASAWRALTKVILPMIKPAIVVALLPNTLIAPKIFDNIYVLTG--GGNN---TGSVSIL  259
MRA RS06565        DGASAWRALTKVILPMIKPAIVVALLPNTLIAPKIFDNIYVLTG--GGNN---TGSVSIL  259
MT RS06535         DGASAWRALTKVILPMIKPAIVVALLPNTLIAPKIFDNIYVLTG--GGNN---TGSVSIL  259
MAF RS06550        DGASAWRALTKVILPMIKPAIVVALLPNTLIAPKIFDNIYVLTG--GGNN---TGSVSIL  259
BQ2027 MB1269      DGASAWRALTKVILPMIKPAIVVALLPNTLIAPKIFDNIYVLTG--GGNN---TGSVSIL  259
BCG RS06710        DGASAWRALTKVILPMIKPAIVVALLPNTLIAPKIFDNIYVLTG--GGNN---TGSVSIL  259
MAV RS06595        DGAGAWRALTKVTLPIIKPAVVVALLPNTLIAPKIFDNIYVLTN--GANN---TGSVSML  264
MAP RS12990        DGAGAWRALTKVTLPIIKPAVVVALLPNTLIAPKIFDNIYVLTN--GANN---TGSVSML  264
OCU RS31270        DGAGPWRALTGVILPIIKPAVVVALLPNTLIAPKIFDNIYVLTN--GANN---TGSVSIL  266
MMAR RS21060       DGAGAWRALTKIILPLIKPAVLVALLPNTLIAPKIFDNIYVLTG--GGND---TASVSIL  266
MAB_1373           DGASGWRALIHVTVPIMKPAILVALLPNTLIAPKIFDNIYVLTG--GGNN---TGSVSIL  250
MSMEG 5060         DGAGPWRKLTKVILPMIKPAILVALLPNTLIAPKIFDNIYILTG--GGND---TGSVSIL  257
ML RS05360         DGAGAWRALTKIILPIIKPAVMVALLPNTLIAPKIFDNIYVLTK--GVNN---TGSVSIL  256
UspA H37Rv         DGASAWQAFWRITLPMLAPTMFFVLVTGIIAAAQVFDTVYALTG--GGPQGS-TDLVAHR  265
MRA RS12305        DGASAWQAFWRITLPMLAPTMFFVLVTGIIAAAQVFDTVYALTG--GGPQGS-TDLVAHR  265
MT RS12160         DGASAWQAFWRITLPMLAPTMFFVLVTGIIAAAQVFDTVYALTG--GGPQGS-TDLVAHR  265
MAF RS12095        DGASAWQAFWRITLPMLAPTMFFVLVTGIIAAAQVFDTVYALTG--GGPQGS-TDLVAHR  265
BQ2027 MB2343      DGASAWQAFWRITLPMLAPTMFFVLVTGIIAAAQVFDTVYALTG--GGPQGS-TDLVAHR  265
BCG RS12065        DGASAWQAFWRITLPMLAPTMFFVLVTGIIAAAQVFDTVYALTG--GGPQGS-TDLVAHR  265
MAV RS09985        DGASAWQAFWRITMPMLAPTTFFVLVTGIVSAAQVFDTVYALTG--GGPAGS-TDLVAHR  269
MAP RS10625        DGASAWQAFWRITMPMLAPTTFFVLVTGIVSAAQVFDTVYALTG--GGPAGS-TDLVAHR  269
OCU RS35040        DGASAWQAFWRITIPMLAPTTFFVLVTGIVSTAQVFDTVYALTG--GGPAGS-TDLVAHR  269
MUL RS06750        DGASAWQAFWRITLPMLAPTTFFVLVTGIVSAAQIFDIVYALTG--GGPKGS-TKLVAHQ  265
MMAR RS18015       DGASAWQAFWRITLPMLAPTTFFVLVTGIVSAAQIFDIVYALTG--GGPKGS-TKLVAHQ  265
MAB 1717c          DGATAWQAFWRITLPMLAPTYFFVLVTGVVSAAQTFDTVYALTA--GGPQGR-TDLAAHR  263
MSMEG 4466         DGADAWQAFWRITLPMLAPTMFFVLVTGIVSAAQVFDTVYALTA--GGPQGR-TDLIAHR  260
ML1768             DGADAWQAFWRITLPMLAPTMFFVLVTGIVSTAQVFDTVYALTD--GGPKGR-TDLVAHR  283
Rv2040c H37Rv      DGASEIRQFVSITVPLIRGALSFVVVISIIHAFQAFDLVYVLTGANGGPKTA-TYVLGIM  252
MRA RS10800        DGASEIRQFVSITVPLIRGALSFVVVISIIHAFQAFDLVYVLTGANGGPKTA-TYVLGIM  252
MT_RS10680         DGASEIRQFVSITVPLIRGALSFVVVISIIHAFQAFDLVYVLTGANGGPKTA-TYVLGIM  252
MAF_RS10635        DGASEIRQFVSITVPLIRGALSFVVVISIIHAFQAFDLVYVLTGANGGPKTA-TYVLGIM  252
BQ2027 MB2066C     DGASEIRQFVSITVPLIRGALSFVVVISIIHAFQAFDLVYVLTGANGGPKTA-TYVLGIM  252
BCG RS10605        DGASEIRQFVSITVPLIRGALSFVVVISIIHAFQAFDLVYVLTGANGGPKTA-TYVLGIM  252
MAV RS11805        DGASEIRQFFAITVPLIRSGISFVVIISIIHAFQAFDMVYVLTGPRGGPKSA-TYVLGIM  266
MAP RS08995        DGASEIRQFFAITVPLIRSGISFVVIISIIHAFQAFDMVYVLTGPRGGPKSA-TYVLGIM  266
OCU RS37010        DGASEIRQFVSITVPLIRSGISFVVIISVIHAFQAFDMVYVLTGANGGPKSA-TYVLGIM  239
MUL RS11900        DGASEIRQFVSITVPLIGSAMSFVVVISIIHAFQAFDLVYVLTGPNGGPKTG-TYVLGIM  269
MMAR RS15035       DGASEIRQFVSITVPLIRSAMSFVVVISIIHAFQAFDLVYVLTGPNGGPKTG-TYVLGIM  269
ML1426             DGASEIRQFISITVPFIRSAISFVVVISFIHAFQTFDLVYVLMGPNGGPKLA-TYVLGIM  271
UgpA H37Rv         DGASRWAVFRRVLLPQLAPTTFFLSITVLINSLQVFDVINVMTK--GGPRDSTTTMVYQ  261
MRA RS15070        DGASRWAVFRRVLLPQLAPTTFFLSITVLINSLQVFDVINVMTK--GGPRDSTTTMVYQ  261
MT RS14970         DGASRWAVFRRVLLPQLAPTTFFLSITVLINSLQVFDVINVMTK--GGPRDSTTTMVYQ  261
MAF RS14760        DGASRWAVFRRVLLPQLAPTTFFLSITVLINSLQVFDVINVMTK--GGPRDSTTTMVYQ  261
BCG RS14700        DGASRWAVFRRVLLPQLAPTTFFLSITVLINSLQVFDVINVMTK--GGPRDSTTTMVYQ  261
MMAR RS09400       DGASRWTSFRRVLLPQLAPTTFFLSITVLINSLQVFDVINVMTK--GGPRDSTTTMVYQ  257
                   **.        : *           :  :  .        :

SugB_H37Rv         NFTG-SSQFEEPTSKIAASAIVITIPIIVF--VLIFGRRIVASLTSGAVKG-          274
MRA RS06570        NFTG-SSQFEEPTSKIAASAIVITIPIIVF--VLIFGRRIVASLTSGAVKG-          274
MT RS06540         NFTG-SSQFEEPTSKIAASAIVITIPIIVF--VLIFGRRIVASLTSGAVKG-          274
MAF RS06555        NFTG-SSQFEEPTSKIAASAIVITIPIIVF--VLIFGRRIVASLTSGAVKG-          274
BQ2027 MB1269      NFTG-SSQFEEPTSKIAASAIVITIPIIVF--VLIFGRRIVASLTSGAVKG-          274
BCG RS06715        NFTG-SSQFEEPTSKIAASAIVITIPIIVF--VLIFGRRIVASLTSGAVKG-          274
MAV RS06600        SFSG-SSQFEEPTSKIAASAVVITVPVILF--VLIFGRRIVASLTSGAVKG-          279
MAP RS12985        SFSG-SSQFEEPTSKIAASAVVITVPIILF--VLIFGRRIVASLTSGAVKG-          279
OCU RS35035        DLQ---SRFNSQWTLVMAATTIAIAPLIVL--FVAFGRRIVASIVVSGLK--          274
OCU RS31275        NFSG-SSQFEEPTSKIAASAVVITVPIIAF--VLIFGRRIVASLTSGAVKG-          279
MUL RS23375        NFAG-SSQFEEPTSKIAASAMVITVPIIVF--VLIFGLRIVASLTSGAVKG-          283
MMAR RS21055       NFAG-SSQFEEPTSKIAASAMVITVPIIVF--VLIFGRRIVASLTSGAVKG-          283
MAB 1374           NFTG-SSQFEEPTSKIAASAIVITVPIIIF--VLIFGRRIVASLTSGAVKG-          277
MSMEG 5059         NFTG-SSQFEEPTSKIAASAMVITIPIIIF--VLIFGRRIVASLTSGAVKG-          278
ML RS05365         NFTG-SSQFEEPTSKIAASAIVITAPIIVF--VMIFGRRIVASLTSGAVKG-          274
UgpE_H37Rv         DLQ---SRFNSQWTLVMAATTVAIVPLIAL--FVTFGRRIVASIVVSGLK--          274
MRA_RS12310        DLQ---SRFNSQWTLVMAATTVAIVPLIAL--FVTFGRRIVASIVVSGLK--          274
MT RS12165         DLQ---SRFNSQWTLVMAATTVAIVPLIAL--FVTFGRRIVASIVVSGLK--          274
MAF RS12100        DLQ---SRFNSQWTLVMAATTVAIVPLIAL--FVTFGRRIVASIVVSGLK--          274
BQ2027 MB2344      DLQ---SRFNSQWTLVMAATTVAIVPLIAL--FVTFGRRIVASIVVSGLK--          274
BCG RS12070        DLQ---SRFNSQWTLVMAATTVAIVPLIAL--FVTFGRRIVASIVVSGLK--          274
MAV RS09980        DLQ---TRFNAQWTLVMAATTIAIVPLVTL--FVIFGRRIVASIVVSGLK--          275
MAP RS10630        DLQ---TRFNAQWTLVMAATTIAIVPLVTL--FVIFGRRIVASIVVSGLK--          275
MUL_RS06745        DLQ---SRFNAQWTLVMAATTVAIVPLIVL--FVAAGRRIVSSIAVSGLK--          279
```

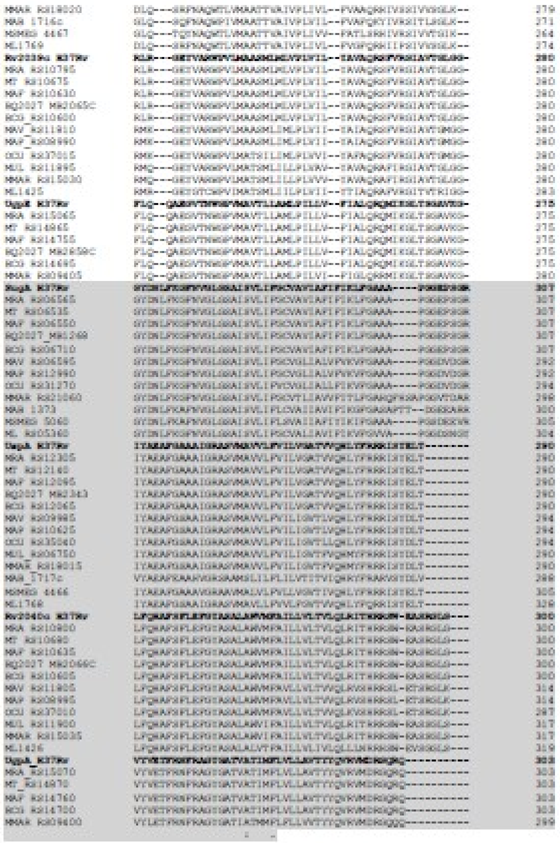

B

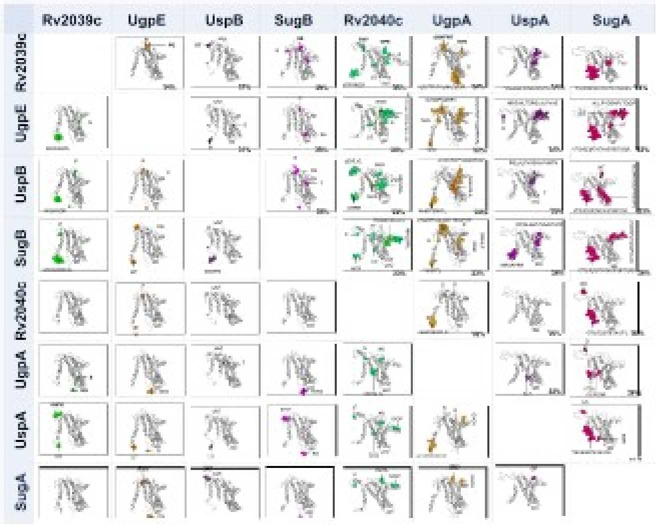

C

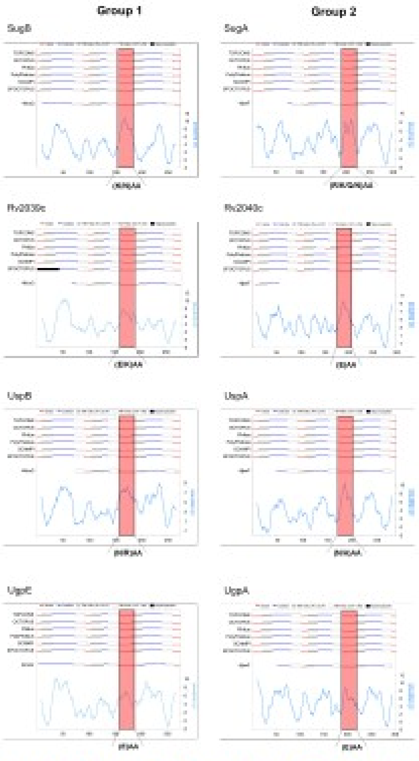

Sequential and structural differences found in the *M. tuberculosis* H37Rv carbohydrate ABC transporters TMDs. A Amino acid sequence alignment of TMDs produced with Clustal Omega program. Proteins can be divided in group 1 (SugB, UspB, UgpB and Rv2039c) and group 2 (SugA, UspA, UgpA and Rv2040c, coloured in gray). The EAA residues of coupling helix are showed in yellow. Residues forming the interface between SugA and SugB that interact with trehalose in the structure of *M. smegmatis* LpqY-SugABC transporter are shown in red. *M. tuberculosis* SugB residues in cyan are the corresponding in *M. smegmatis* SugB forming the scoop loop. **B** Structural comparison of TMDs. Comparison can be made for each two models and the coloured residues represent the position of variable regions between two proteins. The percentage in each box represents the amino acid sequence identity between the two proteins. Models of all proteins were built using SWISS-MODEL program (see Additional file 1, Table S2). **C** Prediction of topology of the TMDs components from *M. tuberculosis*

H37Rv carbohydrate ABC transporters. Amino acid sequences of the proteins were submitted to TOPCONS program. The red bars highlight the position of coupling helices.
