# Supplementary material for: Comparison of carbohydrate ABC importers from Mycobacterium tuberculosis
Source: BMC Genomics. 2021 Nov 19;22:841. doi: 10.1186/s12864-021-07972-w (PMC8603345; doi:10.1186/s12864-021-07972-w)
Supplement: Supplementary file 4 [file 12864_2021_7972_MOESM4_ESM.docx]

Structural and phylogenetic insights of ABC transporters catalysing carbohydrate uptake from *Mycobacterium tuberculosis*

Lilia I. De la Torre, José G. Vergara Meza, Sindy Cabarca Barreto, André G. Costa-Martins, Andrea Balan

Additional File 4

A

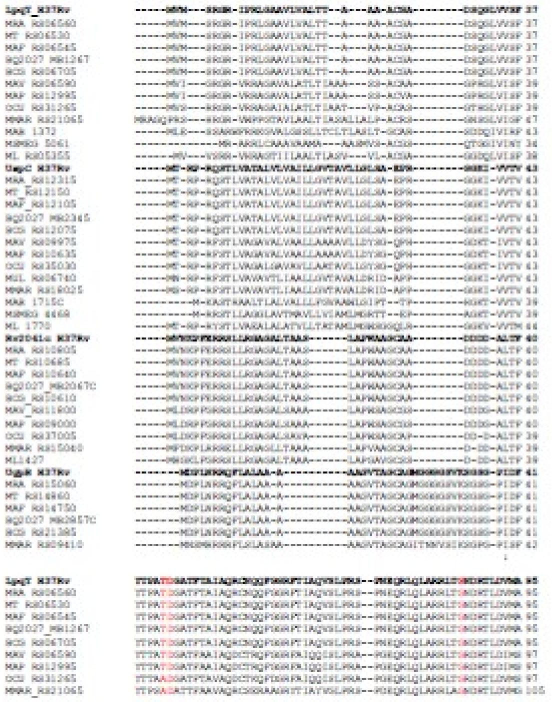

```
MAB 1372            YTPASRAGTFRAAAQRCNRKLGKRPTILQVSLPKR--ADEQRLQLARRLTGRDRTLDLMG 101
MSMEG 5061          YTPANREATFKAVANRCNRQLGKRPQIAQRNLPKR--ADDQRLQLARRLTGRDKSLDVMA 92
ML RS05355          YTPASKDATFTEVARRCTRQFGKRPAIQRVSLPRS--PDEQRLQLARRLTGRDRSLDVMA 96
UspC H37Rv          RL-WDEP--IAAAYRQSFAAPTKRHPDIKVRTNLV--AYSTYFNTLATDVAGHSADDIPW 98
MAA RS12315         RL-WDEP--IAAAYRQSFAAPTKRHPDIKVRTNLV--AYSTYFNTLATDVAGHSADDIPW 98
MT RS12150          RL-WDEP--IAAAYRQSFAAPTKRHPDIKVRTNLV--AYSTYFNTLATDVAGHSADDIPW 98
MAP RS12105         RL-WDEP--IAAAYRQSFAAPTKRHPDIKVRTNLV--AYSTYFNTLATDVAGHSADDIPW 98
Rg2027 MR2345       RL-WDEP--IAAAYRQSFAAPTKRHPDIKVRTNLV--AYSTYFNTLATDVAGHSADDIPW 98
BCS RS12075         RL-WDEP--IAAAYRQSFAAPTKRHPDIKVRTNLV--AYSTYFNTLATDVAGHSADDIPW 98
MAV_RS09975         RV-WGDE--LAAAYRQSFAAPTKAHPDIKVRVNMV--AYSTYFNTLATDVAGHSADDIPW 98
MAP_RS10635         RV-WGDE--LAAAYRQSFAAPTKAHPDIKVRVNMV--AYSTYFNTLATDVAGHSADDIPW 98
OCU_RS35030         RI-WGDQ--IAAAYRQSFQKFSKAHPDIKVRVNLV--AYSTYFNTLATDVAGHSADDIPW 98
MUL RS06740         RL-WAAP--IAAAYQQSFAAPSKTHPNIKVRTNLV--SFSTYFDTLATDVAGHSADDIPW 98
MMAR RS18025        RL-WAAP--IAAAYQQSFAAPSKTHPNIKVRTNLV--SFSTYFDTLATDVAGHSADDIPW 98
MAB 1715C           RV-WDQQ--VAKAYRKSFDKPSKRNPDIQVRVTVT--SYASYFNSLATDVAGHGADDIPW 94
MSMEG 4468          RL-WDPQ--VAAAYRESFDAPSKKHPDIKVRVNTV--AYASYFDSLATDVAGHSADDIPW 94
ML 1770             RL-WADQ--ISTAYRQSFQAPTKTHPDIKVRTNVV--AYSKYFNTLATDVAGHSADDIPW 99
Rv2041c H37Rv       PFRANPD--KLRPRMKVVNKFQRRTPDIKVRALLS--GPGVMQQLATPCAGGKCPDVLM 95
MAA RS10805         PFRANPD--KLRPRMKVVNKFQRRTPDIKVRALLS--GPGVMQQLATPCAGGKCPDVLM 95
MT RS10685          PFRANPD--KLRPRMKVVNKFQRRTPDIKVRALLS--GPGVMQQLATPCAGGKCPDVLM 95
MAP RS10640         PFRANPD--KLRPRMKVVNKFQRRTPDIKVRALLS--GPGVMQQLATPCAGGKCPDVLM 95
Rg2027 MR2067C      PFRANPD--KLRPRMKVVNKFQRRTPDIKVRALLS--GPGVMQQLATPCAGGKCPDVLM 95
BCS RS10610         PFRANPD--KLRPRMKVVNKFQRRTPDIKVRALLS--GPGVMQQLATPCAGGKCPDVLM 95
MAV RS11800         PFRANPE--KRDARMKIIDKPARRHPDIKVRAVLS--GPGVMQQLSTPCVGGKCPDVLM 95
MAP RS09000         PFRANPE--KRDARMKIIDKPARRHPDIKVRAVLS--GPGVMQQLSTPCVGGKCPDVLM 95
OCU RS37005         PFRANPE--KREARMKIIDKPARRHPDIKVRAVLS--GPGVMQQLSTPCVGGKCPDVLM 94
MMAR RS15040        PFRANPE--KADARIKVVDAPSRSHPDIKVRTLLS--GPGALQQISTPCAGGKCPDVLM 94
ML1427              PFRANPE--KTNARMKIVDKPQRRTPDIKVRAVLS--GPGVMQQLATPCAGGKCPDVLM 94
UgpB H37Rv          WSSHPGQ--SRAAKRELIGRFQDRPPTLSVELIDAGKDYDEVAQKFNAALIGTDVPDVVL 99
MAA RS15060         WSSHPGQ--SRAAKRELIGRFQDRPPTLSVELIDAGKDYDEVAQKFNAALIGTDVPDVVL 99
MT RS14960          WSSHPGQ--SRAAKRELIGRFQDRPPTLSVELIDAGKDYDEVAQKFNAALIGTDVPDVVL 99
MAP RS14750         WSSHPGQ--SRAAKRELIGRFQDRPPTLSVELIDAGKDYDEVAQKFNAALIGTDVPDVVL 99
Rg2027 MR2857C      WSSHPGQ--SRAAKRELIGRFQDRPPTLSVELIDAGKDYDEVAQKFNAALIGTDVPDVVL 99
BCS RS21385         WSSHPGQ--SRAAKRELIGRFQDRPPTLSVELIDAGKDYDEVAQKFNAALIGTDVPDVVL 99
MMAR RS09410        WSSHPGQ--SRSVRKVLIDRFQQGPPGLSVELVDAGMDYDEVAKKFNAALIGSDVPDVVV 100
                                                           :            *:.

LpqT H37Rv          LDVVWTAKFAKAGNALPLSEDPA--GLAKNDAVADTLPGFLATAGWNHKLYAAPVTT-NT 152
MAA RS06560         LDVVWTAKFAKAGNALPLSEDPA--GLAKNDAVADTLPGFLATAGWNHKLYAAPVTT-NT 152
MT RS06530          LDVVWTAKFAKAGNALPLSEDPA--GLAKNDAVADTLPGFLATAGWNHKLYAAPVTT-NT 152
MAP RS06545         LDVVWTAKFAKAGNALPLSEDPA--GLAKNDAVADTLPGFLATAGWNHKLYAAPVTT-NT 152
Rg2027 MR1267       LDVVWTAKFAKAGNALPLSEDPA--GLAKNDAVADTLPGFLATAGWNHKLYAAPVTT-NT 152
BCS RS06705         LDVVWTAKFAKAGNALPLSEDPA--GLAKNDAVADTLPGFLATAGWNHKLYAAPVTT-NT 152
MAV RS06590         LDVVWTAKFAKAGNALPLSEDPA--AAKAKADATVDTLPGFLSTARWHDRLPAAPVTT-NT 154
MAP RS12995         LDVVWTAKFAKAGNALPLSEDPA--GRAKADATVDTLPGFLSTARWHDRLPAAPVTT-NT 154
OCU RS31265         LDVVWTAKFAKAGNALPLSEDPA--GRAKPDATADVLPGFLSTARWHDKLPAAPVTT-NT 154
MMAR RS21065        LDVMWTAKFAKAGNALPLSEDPA--GLAKTDATIDTLPGFLATATWKHKLYAAPVTT-NT 162
MAB_1372            MDVVWTAKFAKAGNALPLSEDPA--GVTKAAAQRDALAGFLSDARWKDKLYAAPLST-NT 158
MSMEG 5061          LDVVWTAKFAKAGWAVPLSEDPA--GLAKADATKNTLPGFLATARWQDKLYAAPVTT-NT 149
ML RS05355          MDVVWTAKFAKAGNTLPLSEDPA--GLAKPDAIVDTLPGFLATATWKDKLYAAPVTT-NT 153
UspC H37Rv          LSNAYFAAYADSGRLMKIQT--D---------AADWEKAVVDQFTKGVLWGVPQLTDAG 147
MAA RS12315         LSNAYFAAYADSGRLMKIQT--D---------AADWEKAVVDQFTKGVLWGVPQLTDAG 147
MT RS12150          LSNAYFAAYADSGRLMKIQT--D---------AADWEKAVVDQFTKGVLWGVPQLTDAG 147
MAP RS12105         LSNAYFAAYADSGRLMKIQT--D---------AADWEKAVVDQFTKGVLWGVPQLTDAG 147
Rg2027 MR2345       LSNAYFAAYADSGRLMKIQT--D---------AADWEKAVVDQFTKGVLWGVPQLTDAG 147
BCS RS12075         LSNAYFAAYADSGRLMKIQT--D---------AADWEKAVVDQFTKGVLWGVPQLTDAG 147
MAV RS09975         LSNAYLAAYADSGRLLNIIDTLGTAR------AADWEKPVVEQFTKKGLWGVPQLTDAG 152
MAP RS10635         LSNAYLAAYADSGRLLNIIDTLGTAR------AADWEKPVVEQFTKKGLWGVPQLTDAG 152
OCU RS35030         LSNAYLAAYADSGRLLDIGAALGPTA------AADWEKPVVEQFTKKGTLWGVPQLTDAG 152
MUL RS06740         LSNAYLAAYADSGRLMKIDTAVD---------PGEWEKAVVDQFTKKGVLWGVPQLTDAG 149
MMAR RS18025        LSNAYLAAYADSGRLMKIDTAVD---------PGEWEKAVVDQFTKKGVLWGVPQLTDAG 149
MAB 1715C           LSNAYLSDYADTGNLVPVKP-----------NADWDKPVVAQFTKDGKLWGVPQLSDAG 142
MSMEG 4468          ISNGYFAGYADNGNLLDIADLLGPDA------ATAWEKPVVEQFTKAGALWGVPQLTDAG 148
ML 1770             LSNAYLAAYADNGRLINIGNSLGQPA------TADWEKPVVDQFTKAGALWGVPQLTDAG 153
Rv2041c H37Rv       AWELTTAKLADRSVLLDLNTLLARDQAPAAELKDDSIGALYETFTFNGKQYAFPEQW-SG 154
MAA RS10805         AWELTTAKLADRSVLLDLNTLLARDQAPAAELKDDSIGALYETFTFNGKQYAFPEQW-SG 154
MT RS10685          AWELTTAKLADRSVLLDLNTLLARDQAPAAELKDDSIGALYETFTFNGKQYAFPEQW-SG 154
MAP RS10640         AWELTTAKLADRSVLLDLNTLLARDQAPAAELKDDSIGALYETFTFNGKQYAFPEQW-SG 154
Rg2027 MR2067C      AWELTTAKLADRSVLLDLNTLLARDQVPAAELKDDSIGALYETFTFNGKQYAFPEQW-SG 154
BCS RS10610         AWELTTAKLADRSVLLDLNTLLARDQAPAAELKDDSIGALYETFTFNGKQYAFPEQW-SG 154
MAV RS11800         AWELSTAKLADRSVLLDLGPLLARDKAPAQQLQADSIPALYETFTFNGKQYALPEQW-SG 154
MAP RS09000         AWELSTAKLADRSVLLDLGPLLARDKAPAQQLQADSIPALYETFTFNGKQYALPEQW-SG 154
OCU RS37005         TWELTSAKLADRSVLLDLNPLLARDKAPAQQLKADSIGALYETFAFNGKQYALPEQW-SG 153
MMAR RS15040        AWELSTAKLADRSVFLDLNTMLARDQKFADELAADSIGALYDTFAYNGKQYAFPEQW-SG 153
ML1427              AWDLTTAKLADRSVLLDLNTLLSQDKAPAAELKDDSIKPLYETFTFNGKQYAFPEQW-SG 153
UgpB H37Rv          LDDRWWFKFALSGVLTALDDLFRQVS----VDTTDTVDSLLADYEPNGRKYAVPTAR-ST 154
MAA RS15060         LDDRWWFKFALSGVLTALDDLFRQVS----VDTTDTVDSLLADYEPNGRKYAVPTAR-ST 154
MT RS14960          LDDRWWFKFALSGVLTALDDLFRQVS----VDTTDTVDSLLADYEPNGRKYAVPTAR-ST 154
MAP RS14750         LDDRWWFKFALSGVLTALDDLFRQVS----VDTTDTVDSLLADYEPNGRKYAVPTAR-ST 154
Rg2027_MR2857C      LDDRWWFKFALSGVLTALDDLFRQVS----VDTTDTVDSLLADYEPNGRKYAVPTAR-ST 154
BCS_RS21385         LDDRWWFKFALSGVLTALDDLFRQVS----VDTTDTVDSLLADYEPNGRKYAVPTAR-ST 154
MMAR RS09410        LDDIWWFKFALSGVISPLDKLFRQGS----VDTSDTVDTLLADYEPNGRKYALPTAR-ST 155
                       *  *   :                                   :. *

LpqT H37Rv          QLLWYRPDLVNS-----P-PTDWNAMIA------ER----ARL-------------HAA 182
MAA RS06560         QLLWYRPDLVNS-----P-PTDWNAMIA------ER----ARL-------------HAA 182
MT RS06530          QLLWYRPDLVNS-----P-PTDWNAMIA------ER----ARL-------------HAA 182
MAP_RS06545         QLLWYRPDLVNS-----P-PTDWNAMIA------ER----ARL-------------HAA 182
```

```
Rg2027 MR1267       QLLWYRPDLVNS-----P-PTDNNAMIA------EA----ARL--------------HAA 182
BCG RS06705         QLLWYRPDLVNS-----P-PTDNNAMIA------EA----ARL--------------HAA 182
MAV RS06590         QLLWYRPDLVPQ-----P-PKTNDAVVT------EA----ARL--------------HAA 184
MAP RS12995         QLLWYRPDLVLQ-----P-PKTNDAVVT------EA----ARL--------------HAA 184
OCU RS31265         QLLWYRPDLVRQ-----P-PKTNDGMVR------EA----TRL--------------HAA 184
MMAR RS21065        QLLWYRPDLVAQ-----P-PETNDAVVA------EA----GRL--------------HAA 192
MAB 1372            QLLWYRKDLVPE-----P-PATNDQTVR------EA----RNP--------------AKS 188
MSMEG 5061          QLLWYRADLMPR-----P-PTTNDGMLD------EA----NRL--------------YRR 179
ML RS05355          QLLWYRTDLVDQ-----P-PGDNNGMVA------EA----ARL--------------HAA 183
UmpC_H37Rv          IAVFYNADLLAAAGVDPT-QVD-NLRWDRGED-DTLRRMLAKLTVDADGRTANTPGFDAR 204
MRA_RS12315         IAVFYNADLLAAAGVDPT-QVD-NLRWDRGED-DTLRRMLAKLTVDADGRTANTPGFDAR 204
MT_RS12150          IAVFYNADLLAAAGVDPT-QVD-NLRWDRGED-DTLRRMLAKLTVDADGRTANTPGFDAR 204
MAP RS12105         IAVFYNADLLAAAGVDPT-QVD-NLRWDRGED-DTLRRMLAKLTVDADGRTANTPGFDAR 204
Rg2027 MR2345       IAVFYNADLLAAAGVDPT-QVD-NLRWDRGED-DTLRRMLAKLTVDADGRTANTPGFDAR 204
BCG RS12075         IAVFYNADLLAAAGVDPT-QVD-NLRWDRGED-DTLRRMLAKLTVDADGRTANTPGFDAR 204
MAV RS09975         IALYYNADLLGAAGIDPA-QLA-NLRWDRAGG-DTLRPLLAKLTVDADGNRGDTRGFDPG 209
MAP RS10635         IALYYNADLLGAAGIDPA-QLA-GLRWDPAGG-DTLRPLLAKLTVDADGNRGDTRGFDPG 209
OCU RS35030         IALYYNADLLGAAGVDPA-QLS-TLRWDPDEP-DTLRPVLAKLTVDADGNRGDSRGFDAG 209
MUL RS06740         IAVFYNADLLAAAGVDPA-KLD-GLRWDPGPD-DTLRALLAKLTVDADGRVGATPGFDPG 206
MMAR RS18025        IAVFYNADLLAAAGVDPA-KLD-GLRWDPGPD-DTLRALLAKLTVDADGRVGATPGFDPG 206
MAB 1715C           IALYYNKNLLDAAQVDPA-KLA-KLRWDPDPKVDTLRRMLAKLTAP-------------- 186
MSMEG 4468          IAVYYNADLLEKAGVSPA-DLS-TLRWDRGPD-DTLRPLLAKLTVKEG-----------G 194
ML 1770             IAVFYNADLLTAAGIDPV-QLA-RMQWTRNDD-DTLRPLLTQLTLDTNGRVAKTPGFDSR 210
Rv2041c H37Rv       NFLFYNKQLFDDAGVPPP-PGGNWKRPWDFAEFLDAAQALTKQ--------------GRRG 199
MRA RS10805         NFLFYNKQLFDDAGVPPP-PGGNWKRPWDFAEFLDAAQALTKQ--------------GRRG 199
MT RS10685          NFLFYNKQLFDDAGVPPP-PGGNWKRPWDFAEFLDAAQALTKQ--------------GRRG 199
MAP RS10640         NFLFYNKQLFDDAGVPPP-PGGNWKRPWDFAEFLDAAQALTKQ--------------GRRG 199
Rg2027_MR2067C      NFLFYNKQLFDDAGVPPP-PGGNWKRPWDFAEFLDAAQALTKQ--------------GRRG 199
BCG RS10610         NFLFYNKQLFDDAGVPPP-PGGNWKRPWDFAEFLDAAQALTKQ--------------GRRG 199
MAV RS11800         NTLFYNKKLFDEAGVPSP-PTAWKRPWDFSEFLDTARALTKR--------------DARG 199
MAP RS09000         NTLFYNKKLFDEAGVPSP-PAAWKRPWDFSEFLNAARALTKR--------------DARG 199
OCU RS37005         NTLFYNKKLFADAGVPAP-PTTWQRPWDFSDFLDTARALTKR--------------DTRG 198
MMAR RS15040        NFLFYNKGLFADGGVPPP-PTGWDQPWDFAEFLDTARALTKR--------------DGRG 198
ML1427              NTLFYNKQLFTNAGVQPP-PCTWEQPWDFTEFLDTARALTKR--------------DGRG 198
UgpB H37Rv          FLFYYNKAAWQQAGLFDRGPQSWS-------EP-DGWGPKLQKVVGA----GRGARGWANA 203
MRA RS15060         FLFYYNKAAWQQAGLFDRGPQSWS-------EP-DGWGPKLQKVVGA----GRGARGWANA 203
MT RS14860          FLFYYNKAAWQQAGLFDRGPQSWS-------EP-DGWGPKLQKVVGA----GRGARGWANA 203
MAP RS14750         FLFYYNKAAWQQAGLFDRGPQSWS-------EP-DGWGPKLQKVVGA----GRGARGWANA 203
Rg2027 MR2857C      FLFYYNKAAWQQAGLFDRGLQSWS-------EP-DGWGPKLQKVVGA----GRGARGWANA 203
BCG RS21385         FLFYYNKAAWQQAGLFDRGLQSWS-------EP-DGWGPKLQKVVGA----GRGARGWANA 203
MMAR RS09410        FLFYYNKAVWQRAGLFDRGPNSWQ-------EP-DGWGPKLQKVVDR----RQARGWANA 204
                      .:*.                                  .

LpqY H37Rv          GEPSNIAVGANQSR---GLVYWWPNTLLVSAGGGVLSEDGRRVTLTDTPAARAATVSALQS 239
MRA RS06560         GEPSNIAVGANQSR---GLVYWWPNTLLVSAGGGVLSEDGRRVTLTDTPAARAATVSALQS 239
MT_RS06530          GEPSNIAVGANQSR---GLVYWWPNTLLVSAGGGVLSEDGRRVTLTDTPAARAATVSALQS 239
MAP RS06545         GEPSNIAVGANQSR---GLVYWWPNTLLVSAGGGVLSEDGRRVTLTDTPAARAATVSALQS 239
Rg2027_MR1267       GEPSNIAVGANQSR---GLVYWWPNTLLVSAGGGVLSEDGRRVTLTDTPAARAATVSALQS 239
BCG RS06705         GEPSNIAVGANQSR---GLVYWWPNTLLVSAGGGVLSEDGRRVTLTDTPAARAATVSALQS 239
MAV RS06590         GQPSNIAVGANEGR---GLVYWWPNTLLASGGGRVLSEDGRRVTLTDTPAARAATVNALRS 241
MAP RS12995         GEPSNIAVGANEGR---GLVYWWPNTLLASGGGRVLSEDGRRVTLTDTPAARAATVNALRS 241
OCU RS31265         GQPSNIAVGANEGR---GLVYWWPNTLLASGGGQVLSEDGRRVTLTDTPAARAATVDALRT 241
MMAR RS21065        GQPTNIAVGANQSR---GLMVWWPNTVLSVGGGQVLSEDGTRVTLTDTPAARAATVNALRV 249
MAB 1372            EDPSNIALGSRQTR---GLVYLPNTLLSGAGGGVLAEEGRTVTLTDTPAARQATVTALRT 245
MSMEG 5061          GEPSNIAVGGRQTR---GMVYWWPNTLLQSAGGGVLSDDGQRVTLTDTPEARAATVRALRS 236
ML RS05355          GEPSNIAVGANQSR---GLVYWWPNTLLASAGGGVLSEDGRRVTLTDTPEARAATVRALRS 240
UmpC H37Rv          RVRQWGTNAANDPQ---AIYLMYI--G-SAGSVFQR--DGKFAFDN-----PGAIEAFRY 251
MRA RS12315         RVRQWGTNAANDPQ---AIYLMYI--G-SAGSVFQR--DGKFAFDN-----PGAIEAFRY 251
MT RS12150          RVRQWGTNAANDPQ---AIYLMYI--G-SAGSVFQR--DGKFAFDN-----PGAIEAFRY 251
MAP RS12105         RVRQWGTNAANDPQ---AIYLMYI--G-SAGSVFQR--DGKFAFDN-----PGAIEAFRY 251
Rg2027 MR2345       RVRQWGTNAANDPQ---AIYLMYI--G-SAGSVFQR--DGKFAFDN-----PGAIEAFRY 251
BCG RS12075         RVRQWGTNAANDPQ---AIYLMYI--G-SAGSVFQR--DGKFAFDN-----PGAIEAFRY 251
MAV_RS09975         RVRQWGTNAANDPQ---GIYLMYI--G-SAGSVFQR--GDKFAFDN-----PAAVSAFRY 256
MAP_RS10635         RVRQWGTNAANDPQ---GIYLMYI--G-SAGSVFQR--GDKFAFDN-----PAAVSAFRY 256
OCU_RS35030         RVRQWGTNAANDPQ---GIYLMYI--G-SAGSVFQR--GDKFAFDN-----PGAVTAFQY 256
MUL RS06740         RVRQWGTNAANDPQ---AIYLMYI--G-SAGSVFRR--DNKFAFDN-----PPAIDAFRY 253
MMAR RS18025        RVRQWGTNAANDPQ---AIYLMYI--G-SAGSVFRR--DNKFAFDN-----PPAIDAFRY 253
MAB 1715C           --GRWGTNAANDLQ---GIYLMYL--G-SAGAVFQR--GDKFAFAK-----PRAEMAFTY 231
MSMEG 4468          RTRQWGTNAANDLQ---GIYLMFI--G-SAGSTFSE--GDRFTYDN-----PQAVEAFRY 241
ML 1770             RVRQWGTNAANDPQ---AIYLMYI--G-SAGSVFQR--GDKFAFDN-----PAAVEAFRY 257
Rv2041c H37Rv       KDRQWGSFVNAWNSFYAAGLFAMNN--GVPWSVRWR--PTKLNFDN-----DGFLEAVQF 250
MRA RS10805         KDRQWGSFVNAWNSFYAAGLFAMNN--GVPWSVRWR--PTKLNFDN-----DGFLEAVQF 250
MT RS10685          KDRQWGSFVNAWNSFYAAGLFAMNN--GVPWSVRWR--PTKLNFDN-----DGFLEAVQF 250
MAP RS10640         KDRQWGSFVNAWNSFYAAGLFAMNN--GVPWSVRWR--PTKLNFDN-----DGFLEAVQF 250
Rg2027 MR2067C      KDRQWGSFVNAWNSFYAAGLFAMNN--GVPWSVRWR--PTKLNFDN-----DGFLEAVQF 250
BCG RS10610         KDRQWGSFVNAWNSFYAAGLFAMNN--GVPWSVRWR--PTKLNFDN-----DGFLEAVQF 250
MAV RS11800         RAAQYGPVNTWNSYYSAGLFAMNN--GVPWSDPRLN--PTHPNFDN-----AAPQEAVQF 250
MAP RS09000         RAAQYGPVNTWNSYYSAGLFAMNN--GVPWSDPRLN--PTHPNFDN-----AAPQEAVQF 250
OCU RS37005         RAAQYGPVNTWNSYTSAGLFAMNN--GVPWSNPRLN--PTHLNFDD-----AAPQEAVQF 249
MMAR_RS15040        RVTQWGPVDTPVPYLSAGLFALNN--GVPWSTVRWR--PTKLNFDD-----DAPIEAVQF 249
ML1427              RVTQWGPVNTNLSYYTAGLFALNN--GVPWSNVRWR--PTKLNFDD-----DAPIEAVQF 249
UgpB H37Rv          DLISWTFQGPNW------------------APGGAYSD--KWTLTLTR-----PATIAAGNF 240
MRA RS15060         DLISWTFQGPNW------------------APGGAYSD--KWTLTLTR-----PATIAAGNF 240
MT RS14860          DLISWTFQGPNW------------------APGGAYSD--KWTLTLTR-----PATIAAGNF 240
MAP RS14750         DLISWTFQGPNW------------------APGGAYSD--KWTLTLTR-----PATIAAGNF 240
Rg2027 MR2857C      DLISWTFQGPNW------------------APGGAYSD--KWTLTLTR-----PATIAAGNF 240
BCG RS21385         DLISWTFQGPNW------------------APGGAYSD--KWTLTLTR-----PATIAAGNF 240
MMAR_RS09410        ELISWTFQGVNW------------------TPGGAYSD--LWTLAPTD-----PGTIAAGNF 241
```

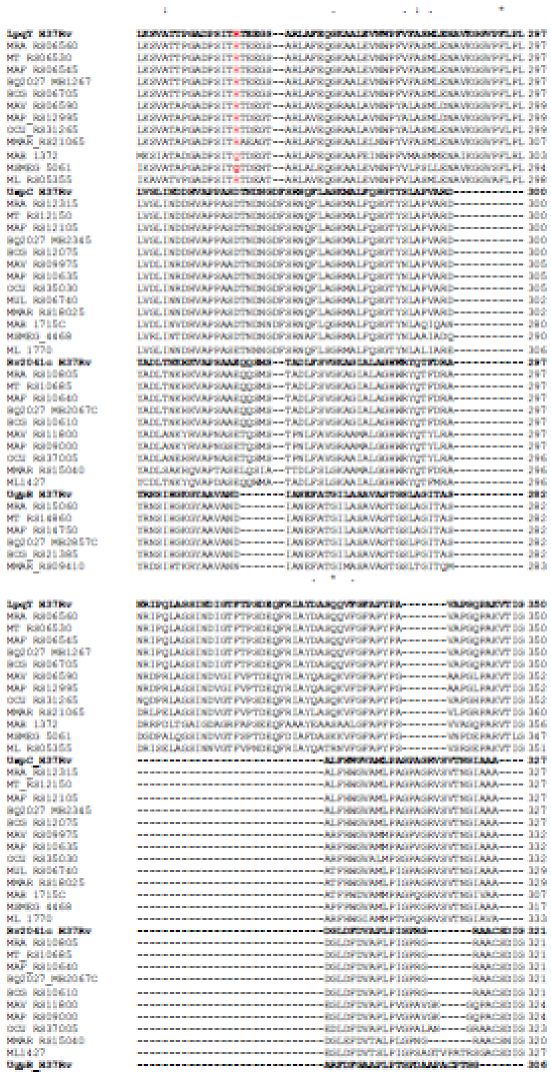

LpqT H37Rv
UspC H37Rv
Rv2041c H37Rv
UgpB H37Rv

```
MAA Rs15060           ------------------------------ARFDPGAAPLPTSRDAAPACPTSG------- 306
MT Rs14860            ------------------------------ARFDPGAAPLPTSRDAAPACPTSG------- 306
MAP Rs14750           ------------------------------ARFDPGAAPLPTSRDAAPACPTSG------- 306
Bg2027 MB2857C        ------------------------------ARFDPGAAPLPTSRDAAPACPTSG------- 306
BCS Rs21385           ------------------------------ARFDPGAAPLPTSRDAAPACPTSG------- 306
MMAR Rs09410          ------------------------------ARFDPGAAPVPTSRDGAPGCPTSG------- 307
                                                         . :  *

LpqT H37Rv            GLNLAVR--KTTKHRARAFKAVKCLADQHNQKYVSLKGGLPAVRASLYSDPQ---FQAKY 605
MAA Rs06560           GLNLAVR--KTTKHRARAFKAVKCLADQHNQKYVSLKGGLPAVRASLYSDPQ---FQAKY 605
MT Rs06530            GLNLAVR--KTTKHRARAFKAVKCLADQHNQKYVSLKGGLPAVRASLYSDPQ---FQAKY 605
MAP Rs06545           GLNLAVR--KTTKHRARAFKAVKCLADQHNQKYVSLKGGLPAVRASLYSDPQ---FQAKY 605
Bg2027 MB1267         GLNLAVR--KTTKHRARAFKAVKCLADQHNQKYVSLKGGLPAVRASLYSDPQ---FQAKY 605
BCS Rs06705           GLNLAVR--KTTKHRARAFKAVKCLADQHNQKYVSLKGGLPAVRASLYSDPQ---FQAKY 605
MAV Rs06590           GANLAVR--STTKHRARAFKAIKCVRSLQHQKYVAIQGGLPPVRTSLYSDPQ---FQTKY 607
MAP Rs12995           GANLAVR--STTKHRARAFKAIKCVRSLQHQKYVAIQGGLPPVRTSLYSDPQ---FQTKY 607
OCU Rs31265           GANLAVR--STTKHRARAFKAIKCLASLQHQKYVSIAGGLPPVRTSLYSDPQ---FQAKY 607
MMAR Rs21065          GLNLAVR--STSKHKARAFKAVKCLADPQSQKKLAIKGGLPAVRTSLYADPQ---FQAKY 615
MAB 1372              GLNIAVR--KTTKYRAQAFKALAKCLASKKNQKATAIKGGLPAVDSSLYSDPE---FQAKY 611
MSMEG 5061            GLNLAVR--STSQHKARAFKAIKCLAHVKNQKYTSIKGGLPAVRTSLYSDPA---FQKKY 602
ML Rs05355            GLNLAVR--STSKHKARAFKAVKCLASKQSQQYLAIKGGLPAVRASLYSDPA---FQDKY 606
UspC H37Rv            --GN-----AASKHPDAVRQVLAMMDSTKGNSYLGRHGAAIPAVLSAQPVYP--DYNAA- 377
MAA Rs12315           --GN-----AASKHPDAVRQVLAMMDSTKGNSYLGRHGAAIPAVLSAQPVYP--DYNAA- 377
MT Rs12150            --GN-----AASKHPDAVRQVLAMMDSTKGNSYLGRHGAAIPAVLSAQPVYP--DYNAA- 377
MAP Rs12105           --GN-----AASKHPDAVRQVLAMMDSTKGNSYLGRHGAAIPAVLSAQPVYP--DYNAA- 377
Bg2027 MB2365         --GN-----AASKHPDAVRQVLAMMDSTKGNSYVGRHGAAIPAVLSAQPVYP--DYNAA- 377
BCS Rs12075           --GN-----AASKHPDAVRQVLAMMDSTKGNSYVGRHGAAIPAVLSAQPVYP--DYNAA- 377
MAV Rs09975           --GN-----AKTKHPGAVRQVLAMMDSKQGNKYLGKYGAAIPAVTSAQPVYP--DYNAA- 382
MAP Rs10635           --GN-----AKTKHPAAVRQVLAMMDSKQGNKYLGKYGAAIPAVTSAQPVYP--KYNAS- 382
OCU Rs35030           --GN-----AASKHPDAVRKVLAMMDSKQGNKYLGKYGAAIPAVSSAQPVYP--KYNAT- 382
MUL Rs06740           --GN-----SASQHPDAVRQVLAMMDSSAGNKYLGKDVAIPAVKSAQPSYP--AFNEA- 379
MMAR Rs18025          --GN-----SASQHPDAVRQVLAMMDSSAGNKYLGKDVAIPAVKSAQPSYP--AFNEA- 379
MAB 1715C             --AN-----SSSPHPDAVRKVLAMMDSTDGNSFLGKSGAAIPAVLSARAPYP--QYNAD- 357
MSMEG 4469            --GN-----AKTKHPEAVRKVLAMMDSKRGNKFVGKHGAAIPAVLAAQPVYH--KYNAS- 367
ML 1770               --GN-----AKTKHPDAVRQVLAMMDSKKGNAYLGKHGAAVPAVKSAQPSYP--DYNAA- 383
Rv2041c H37Rv         VTSLAIA--ATSKKKDQAMKFVKFATSPVGQALIGESKLFVPVLRSAINSKG--FANAS- 376
MAA Rs10805           VTSLAIA--ATSKKKDQAMKFVKFATSPVGQALIGESKLFVPVLRSAINSKG--FANAS- 376
MT Rs10685            VTSLAIA--ATSKKKDQAMKFVKFATSPVGQALIGESKLFVPVLRSAINSKG--FANAS- 376
MAP Rs10640           VTSLAIA--ATSKKKDQAMKFVKFATSPVGQALIGESKLFVPVLRSAINSKG--FANAS- 376
Bg2027 MB2067C        VTSLAIA--ATSKKKDQAMKFVKFATSPVGQALIGESKLFVPVLRSAINSKG--FANAS- 376
BCS Rs10610           VTSLAIA--ATSKKKDQAMKFVKFATSPVGQALIGESKLFVPVLRSAINSKG--FANAS- 376
MAV Rs11800           ATSLAIS--SSSPKKEQAMKFVKFATSPVGQALIGESCLFVPVLRSALKSDG--FANAS- 379
MAP Rs09000           ATSLAIS--SSSPKKEQAMKFVKFATSPVGQALIGESCLFVPVLRSALKSDG--FANAS- 379
OCU Rs37005           ATSLGIS--STSPKKDQAMKFVKFATSPVGQALIGESCLFVPVLRSALKSDG--FANAS- 378
MMAR Rs15040          ATSLAIA--ASSAKKEQAMKFVKFATSPAGQALIGETSLFVPVLGSALASAG--FAKAS- 375
ML1427                ATSLAIA--ASSSKKEQAMKFVKFATSPAGQALIGESCLFVPVLQSAITSTG--FAKAS- 382
UgpB_H37Rv            -ASLAIPAKLSEEKKVNALKFIAFVTNPTNTAYFSQQTGYLPVRKSAVDDASKKKYLADN 365
MAA_Rs15060           -ASLAIPAKLSEEKKVNALKFIAFVTNPTNTAYFSQQTGYLPVRKSAVDDASKKKYLADN 365
MT Rs14860            -ASLAIPAKLSEEKKVNALKFIAFVTNPTNTAYFSQQTGYLPVRKSAVDDASKKKYLADN 365
MAP Rs14750           -ASLAIPAKLSEEKKVNALKFIAFVTNPTNTAYFSQQTGYLPVRKSAVDDASKKKYLVDN 365
Bg2027 MB2857C        -ASLAIPAKLSEEKKVNALKFIAFVTNPTNTAYFSQQTGYLPVRKSAVDDASKKKYLADN 365
BCS Rs21385           -ASLAIPAKLSEEKKVNALKFIAFVTNPTNTAYFSQQTGYLPVRKSAVDDASKKKYLADN 365
MMAR Rs09410          -ASLAIPTKLSDKKKANALKFIKYITNPVNTAYFSQKTGYLAVRKSAADVPKKQKYLADN 366
                               :     . . :    .          .       .  :

LpqT H37Rv            ---------PMAAIIKQGLTDAAVRKATPVTQALSIRLAAVLSPITKID--PKSTA----- 650
MAA Rs06560           ---------PMAAIIKQGLTDAAVRKATPVTQALSIRLAAVLSPITKID--PKSTA----- 650
MT Rs06530            ---------PMAAIIKQGLTDAAVRKATPVTQALSIRLAAVLSPITKID--PKSTA----- 650
MAP Rs06545           ---------PMAAIIKQGLTDAAVRKATPVTQALSIRLAAVLSPITKID--PKSTA----- 650
Bg2027 MB1267         ---------PMAAIIKQGLTDAAVRKATPVTQALSIRLAAVLSPITKID--PKSTA----- 650
BCS Rs06705           ---------PMAAIIKQGLTDAAVRKATPVTQALSIRLAAVLSPITKID--PKSTA----- 650
MAV Rs06590           ---------PMTTIIKKQLTDAAVRKATPVTQTVSIRLAATLSPITSID--PKSTA----- 652
MAP Rs12995           ---------PMTTIIKKQLTDAAVRKATPVTQTVSIRLAATLSPITSID--PKSTA----- 652
OCU_Rs31265           ---------PMTTIIKKQLTDAAVRKATPVTQAVAIRLAATLSPITKID--PKSTA----- 652
MMAR_Rs21065          ---------PMTKIIKQGLTKAAVRKATPVTQAVSILALAAELSPVTKID--PKSTA----- 660
MAB 1372              ---------PATAIIKDQLAKAAVRKASPYTQAISTRVSAVLAPITSID--PKSTA----- 656
MSMEG 5061            ---------PQTEIIKQGLTNAAVRKATPVTQAVSTRMSATLAPISDID--PKSTA----- 647
ML Rs05355            ---------PMTTIIKQGLTDAAVRPVTPATQAVSIRLSAALSPITDID--PKPMA----- 651
UspC H37Rv            --RSVINTPFFKAVLNGPRIA---APKSAGFAAKQGALKPTFDKMFLGKSDVTTTLKQAQA 432
MAA Rs12315           --RSVINTPFFKAVLNGPRIA---APKSAGFAAKQGALKPTFDKMFLGKSDVTTTLKQAQA 432
MT Rs12150            --RSVINTPFFKAVLNGPRIA---APKSAGFAAKQGALKPTFDKMFLGKSDVTTTLKQAQA 432
MAP Rs12105           --RSVINTPFFKAVLNGPRIA---APKSAGFAAKQGALKPTFDKMFLGKSDVTTTLKQAQA 432
Bg2027 MB2345         --RSVINTPFFKAVLNGPRIA---APKSAGFAAKQGALKPTFDKMFLGKSDVTTTLKQAQA 432
BCS Rs12075           --RSVINTPFFKAVLNGPRIA---APKSAGFAAKQGALKPTFDKMFLGKSDVTTTLKQAQA 432
MAV Rs09975           --RSVINTPFFKAVLNGPRIA---APKSAGFAAGNDALAPTFDKMFSGKSDVATTLKKAQA 437
MAP Rs10635           --RSVINTPFFKAVLNGPRIA---APKSAGFAAGNDALAPTFDKMFSGKSDVATTLKKAQA 437
OCU Rs35030           --RSVINTPFFTVLNGPKIA---APKSAGFAAGNDALQPTFDKMFLGKSDVAVTLKKAQA 437
MUL Rs06740           --KSVNVNKPFFKAVLSGSKIP---APKSAGFAAGNDALKPTFDKMFLGKQVATILKKAQA 434
MMAR Rs18025          --KSVNVNKPFFKAVLSGSKIP---APKSAGFAAGNDALKPTFDKMFLGKQVATILKKAQA 434
MAB 1715C             --RSVINSKPFFKVLAKQQIA---APKSQGPGAGPAALKPTFAKMFLGKLDVKKALQQAQK 412
MSMEG 4469            --RSVINSKPFFKVLQGPKIA---APKSAGFPAGPEALTPTFAKMFLGKKDVAGTLKKAQK 422
ML 1770               --KSIINTPFFKVLDGPKIP---APKSAGFPAGDDALQPTFDKMFLGKSDVKKILQQAQA 438
Rv2041c H37Rv         --RKV--GNLKVLSKSPAYS-KSLPVTPAWEKIAALMDKTFNPVLKGSKPATSLTGLS-Q 430
MAA Rs10805           --RKV--GNLKVLSKSPAYS-KSLPVTPAWEKIAALMDKTFNPVLKGSKPATSLTGLS-Q 430
MT Rs10685            --RKV--GNLKVLSKSPAYS-KSLPVTPAWEKIAALMDKTFNPVLKGSKPATSLTGLS-Q 430
MAP Rs10640           --RKV--GNLKVLSKSPAYS-KSLPVTPAWEKIAALMDKTFNPVLKGSKPATSLTGLS-Q 430
Bg2027 MB2067C        --RKV--GNLKVLSKSPAYS-KSLPVTPAWEKIAALMDKTFNPVLKGSKPATSLTGLS-Q 430
BCS_Rs10610           --RKV--GNLKVLSKSPAYS-KSLPVTPAWEKIAALMDKTFNPVLKGSKPATSLTGLS-Q 430
```

```
MAV RS11800          --RRV--GNLGVLTDGPAFS-QSLPITPAWEKVNALMDRNPSPILRGSRPATSLAGLS-R 433
MAP RS09000          --RRV--GNLGVLTDGPAFS-QSLPITPAWEKVNALMDRNPSPILRGSRPATSLAGLS-R 433
OCU RS37005          --RRI--GNLGVLTKGPAYS-QSLPITPAWEKVNALIDRNPSPVLRGRQPATSLAGLS-R 432
MMAR RS15060         --RRV--DNLAVLTAGPDHS-QSLPITPAWPKVNALMDRNPSPVLRGSRPATSLIGMS-R 429
ML1427               --RRV--ANLAVLTDGPVHS-AGLPITPAWEKINALMDRNPSPVLRGVRPATSLAGLA-R 436
UgpB H37Rv           PRARVALDQLPWT-RTQDTARVFLPGSD-------RIISAGLESIGLRGADVTKTPTNIQK 418
MRA RS15060          PRARVALDQLPWT-RTQDYARVFLPGSD-------RIISAGLESIGLRGADVTKTPTNIQK 418
MT RS14860           PRARVALDQLPWT-RTQDYARVFLPGSD-------RIISAGLESIGLRGADVTKTPTNIQK 418
MAF RS14750          PRARVALDQLPWT-RTQDYARVFLPGSD-------RIISAGLESIGLRGADVTKTPTNIQK 418
BQ2027_MB2857C       PRARVALDQLPWT-RTQDYARVFLPGSD-------RIISAGLESIGLRGADVTKTPTNIQK 418
BCG_RS21385          PRARVALDQLPWT-RTQDYARVFLPGSD-------RIISAGLESIGLRGADVTKTPTNIQK 418
MMAR_RS09410         PRARVAINQLPWT-RPQDYARVFLPGAD-------RIISAGLESIGLRGTNVAKTPASIER 419
                                                *           :   :  :

LpqY H37Rv           ---DKLAAQAQKAIDGMRLLP         468
MRA RS06540          ---DKLAAQAQKAIDGMRLLP         468
MT RS06530           ---DKLAAQAQKAIDGMRLLP         468
MAF RS06545          ---DKLAAQAQKAIDGMRLLP         468
BQ2027 MB1267        ---DKLAAQAQKAIDGMRLLP         468
BCG RS06705          ---DKLAAQAQKAIDGMRLLP         468
MAV RS06590          ---DQLAAKVQKAIDGRSLLP         470
MAP RS12995          ---DQLAVKVQKAVDGRSLLP         470
OCU RS31265          ---DKLAAKVQKAIDGRSLLP         470
MMAR RS21065         ---DKLAAQVQQAIDGRSLLP         478
MAB 1372             ---DLLADQVQKAVDGRSLIP         474
MSMEG 5061           ---DKLTKAVQKAIDGRSLIP         465
ML RS05355           ---DKLAAQVQKAIDGRSLLP         469
UspC_H37Rv           AANAATQR-------------         440
MRA RS12315          AANAATQR-------------         440
MT RS12150           AANAATQR-------------         440
MAF RS12105          AANAATQR-------------         440
BQ2027 MB2345        AANAATQR-------------         440
BCG RS12075          AANAATQR-------------         440
MAV RS09975          AANAAAARR------------         446
MAP RS10635          AANAAARR-------------         445
OCU RS35030          AANAAAARQ------------         446
MUL RS06740          AANAAARR-------------         442
MMAR RS18025         AANAAARR-------------         442
MAB 1715C            AANKALER-------------         430
MSMEG 4468           AANAAASR-------------         430
ML 1770              AANTAARR-------------         446
Rv2041c H37Rv        AVDEVLRSP------------         439
MRA RS10605          AVDEVLRSP------------         439
MT RS10685           AVDEVLRSP------------         439
MAF RS10640          AVDEVLRSP------------         439
BQ2027_MB2067C       AVDEVLRSP------------         439
BCG_RS10610          AVDEVLRSP------------         439
MAV_RS11800          SVDEVLRSP------------         442
MAP RS09000          SVDEVLRSP------------         442
OCU RS37005          SVDEVLRSP------------         441
MMAR RS15060         ALDEVLRSP------------         438
ML1427               AVDEVLRSP------------         445
UgpB H37Rv           RLQVILDRQIMRKLAGRS---         436
MRA RS15060          RLQVILDRQIMRKLAGRS---         436
MT RS14860           RLQVILDRQIMRKLAGRS---         436
MAF RS14750          RLQVILDRQIMRKLAGRS---         436
BQ2027 MB2857C       RLQVILDRQIMRKLAGRS---         436
BCG RS21385          RLQVILDRQIMRKLAGRS---         436
MMAR_RS09410         QLQIILDRQIVRKLAQRS---         437
```

B

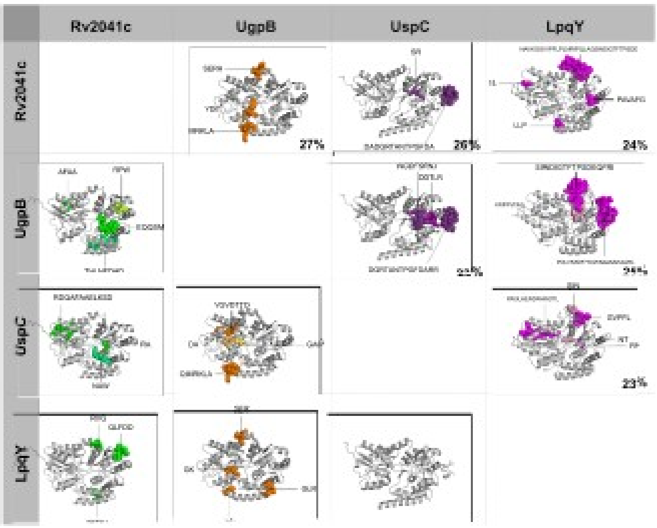

Sequential and structural position of the main differences between the *M. tuberculosis* sugar transporters SBPs. **A** Amino acid sequence alignment of the SBPs showing the four groups (bold letters). The alignment was made using Clustal Omega. Residues in red are involved in the ligand binding. **B** Proteins in cartoon represent the modelled structures of all SBPs, except for UgpB and UspC that have crystallographic structures (PDB IDs: 4MFI and 5K2X, respectively). After the amino acid sequence alignment of each two structures different regions were showed by coloured spheres and can be compared. Models of LpqY and Rv2041c were built using SWISS-MODEL program [37] (see Additional file 1, Table S2).
