# Supplementary material for: Comparison of carbohydrate ABC importers from Mycobacterium tuberculosis
Source: BMC Genomics. 2021 Nov 19;22:841. doi: 10.1186/s12864-021-07972-w (PMC8603345; doi:10.1186/s12864-021-07972-w)
Supplement: Supplementary file 5 [file 12864_2021_7972_MOESM5_ESM.docx]

Structural and phylogenetic insights of ABC transporters catalysing carbohydrate uptake from *Mycobacterium tuberculosis*

Lilia I. De la Torre, José G. Vergara Meza, Sindy Cabarca Barreto, André G. Costa-Martins, Andrea Balan

Additional File 5

A

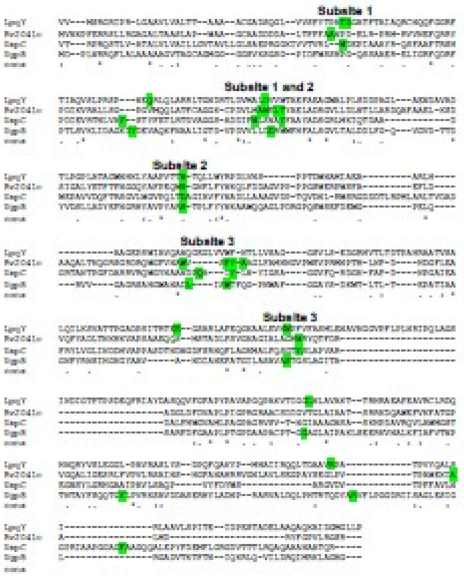

B

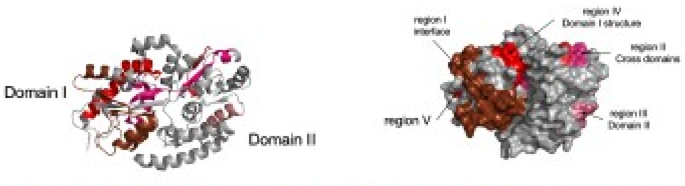

Amino acid sequence alignment of the *M. tuberculosis* H37Rv sugar binding proteins and structural information. A Alignment of UgpB, Rv2041c, LpqY and UspC obtained with Expresso program in the T-coffee server (http://tcoffee.crg.cat/apps/tcoffee/do:expresso) Amino acids that form the ligand-binding pocket are highlighted in green. The region of the three subsites is showed. B Three-dimensional structure of UgpB in cartoon showing the five regions with highest conservation (left). The putative function of each region is represented in the structure in color surface (right).
